# The Role of the OSM/OSMRβ Axis in Chronic Liver Disease Progression and Hepatocellular Carcinoma Development: A Focus on MASLD/MASH

**DOI:** 10.3390/ijms27188213

**Published:** 2026-09-15

**Authors:** Beatrice Foglia, Jessica Nurcis, Marta Signorini, Erica Novo, Claudia Bocca, Stefania Cannito, Maurizio Parola

**Affiliations:** Unit of Experimental Medicine and Clinical Pathology, Department of Clinical and Biological Sciences, University of Torino, Corso Raffaello 30, 10125 Torino, Italy; beatrice.foglia@unito.it (B.F.); jessica.nurcis@unito.it (J.N.); marta.signorini@edu.unito.it (M.S.); erica.novo@unito.it (E.N.); claudia.bocca@unito.it (C.B.)

**Keywords:** MASH, OSM/OSMRβ axis, chronic inflammation, liver fibrosis, hepatocellular carcinoma

## Abstract

Metabolic dysfunction-associated steatotic liver disease (MASLD) represents the emerging leading cause of Chronic Liver Disease (CLD) worldwide, with a global prevalence of approximately 30% in the general population that parallels global rates of obesity and Type 2 Diabetes (T2D). Although two drugs (Resmetirom and Semaglutide) have been recently approved for clinical use, their ability to block or slow down disease progression to metabolic dysfunction-associated Steatohepatitis (MASH) and liver fibrosis is limited to a subset of patients, and no data are available at present for the efficacy of these drugs in compensated cirrhosis or in relation to hepatocellular carcinoma (HCC) development. Moreover, there is a lack of reliable biomarkers able to identify MASH patients at risk of disease progression and/or HCC development. In line with the knowledge that pro-inflammatory cytokines play a key role in MASLD/MASH progression and HCC development, in this review, we will discuss the role in this disease and other CLDs of Oncostatin M (OSM), a cytokine belonging to the IL6 family, and of pathways involving OSM and its receptor β (OSM/OSMRβ axis). OSM and related pathways are emerging as selective in sustaining disease progression by promoting chronic inflammation and fibrogenesis. The OSM/OSMRβ axis is proposed to be involved in MASH-related HCC development by affecting proliferation, angiogenesis, invasiveness and metastasis as well as by reshaping the MASH-related tumor immune microenvironment. OSM and the OSM/OSMRβ axis are emerging as a candidate biomarker and putative MASH-related therapeutic target.

## 1. Metabolic Dysfunction-Associated Steatotic Liver Disease (MASLD): A Relevant Disease Worldwide

Metabolic dysfunction-associated steatotic liver disease (MASLD) represents the emerging leading cause of Chronic Liver Disease (CLD) worldwide, with epidemiological studies indicating that MASLD affects approximately 1 billion individuals globally (including adults, children and adolescents) in the general population [1]. A recent meta-analysis performed by pooling MASLD prevalence estimates and ultrasound-defined MASLD revealed an overall global prevalence of approximately 30.0% [1]; moreover, in the USA, Europe and Southeast Asia, MASLD prevalence is predicted to further rise by 2030, affecting more than 400 million individuals in these areas [2]. Such a high global prevalence of MASLD is expected to coincide with rising rates of obesity and Type 2 Diabetes (T2D) worldwide, with approximately 65% of T2D patients having MASLD. This metabolic disease is currently viewed as the hepatic manifestation of metabolic syndrome and is associated with additional metabolic risk factors [3]. MASLD can be diagnosed on the basis of the following major evidence: hepatic steatosis upon histopathological analysis of liver biopsies, or imaging techniques indicating the presence of more than 5% of hepatocytes with an accumulation of triglycerides (TGs) in association with at least one or more cardiometabolic risk factors (including altered levels of body mass index (BMI), fasting glucose, blood pressure, plasma triglycerides, or plasma HDL/cholesterol). Diagnosis of MASLD also requires that these patients consume little or no alcohol, and that the involvement of other metabolic, hereditary, toxic- or drug-induced causes of fatty liver can be excluded [4].

### 1.1. MASLD as a Progressive Chronic Liver Disease: From Simple Steatosis to Cirrhosis and Hepatocellular Carcinoma (HCC) Development

MASLD, according to histopathological analyses, is currently defined as a dynamic spectrum of conditions ranging from simple steatosis to the progressive form of metabolic dysfunction-associated steatohepatitis (MASH) [5]. Approximately 75–80% of MASLD patients present only simple steatosis, which is usually considered a non-progressive or slowly progressive condition, although paired-biopsy longitudinal studies have shown that a subset of patients with simple steatosis can progress to MASH and advanced fibrosis [6]. The diagnosis of MASH implies morphological evidence of steatosis associated with aspects of hepatocyte injury and death (including ballooning, apoptosis and other kinds of cell death such as necroptosis, pyroptosis and ferroptosis) as well as lobular and/or portal inflammatory infiltrate and a variable degree of fibrosis. MASH patients are at risk of fibrogenic progression towards a more advanced stage of CLD and cirrhosis; although the natural course of MASLD is inconstant and characterized by bidirectional and concordant changes in both disease activity and fibrosis stage, MASH-associated liver fibrosis is considered the strongest predictor for disease-specific progression and mortality [6]. Finally, cirrhotic MASH patients carry a 1.3–2% per year risk of developing hepatocellular carcinoma (HCC), and overall MASH is currently indicated as the most rapidly growing cause of HCC in liver transplant candidates [7,8].

HCC accounts for approximately 85–90% of all primary liver cancers, representing the sixth most common cancer and the third leading cause of cancer-related death worldwide. HCC is often diagnosed at an advanced stage in most patients (50–60%), with survival at five years from diagnosis usually less than 30% due to the insufficient results obtained with conventional therapeutic approaches (i.e., surgical resection or, for unresectable tumors, transarterial chemoembolization (TACE), or systemic treatments), with some improvement more recently related to the use of immune-checkpoint inhibitors (ICIs) in viral patients [1,5,7,8,9,10,11]. Unfortunately, the overall scenario is even more unsatisfying for MASLD/MASH patients. In addition to the very high prevalence of MASLD in the general population, one should consider the following: (i) Unlike other etiologies, in which HCC mainly develops in cirrhotic patients, MASH-related HCC is now increasingly reported in non-cirrhotic patients [8,9]. (ii) At present, only two drugs, Resmetirom and Semaglutide, have been conditionally approved for the treatment of MASH patients with F2/F3 fibrosis; both drugs were able to reduce fibrosis and led to MASH resolution in a subset of patients (with Semaglutide being more efficient than Resmetirom), but whether they may be clinically useful in compensated cirrhosis or against HCC development is still unknown (see ref. [12] for a review dedicated to emerging therapies for MASLD/MASH). (iii) Other persisting challenges are represented by the lack of validated biomarkers able to identify MASH patients at risk of disease progression and/or HCC development, ongoing reliance on biopsy-based surrogates, high placebo response rates and heterogeneity in disease biology and clinical course. (iv) Available data suggest that ICIs may be less efficient in MASLD/MASH-related HCC than for hepatic tumors of different etiology (particularly vs. HCC patients with viral etiology) [13]; along these lines, murine studies of MASH-HCC reported a lack of efficacy of ICIs that was related to changes in tumor microenvironment (i.e., decrease in antitumor CD4+ and CD8+ T cells, exhaustion of PD-1+ CD8+ T cells, aberrant CD8+ T-cell activation within the tumor exerting cytotoxicity vs. hepatocytes). However, data from clinical cohorts show mixed findings in relation to ICI efficacy in MASH-HCC, and caution is needed when extrapolating preclinical data to MASH patients [13,14].

### 1.2. The Metabolic and Pathophysiological Basis of MASLD/MASH: Of Fatty Acid, Carbohydrate and Liver X Receptors (LXR)/Cholesterol Metabolism

MASLD is closely linked to obesity and T2D [3,6,10,15], and the resulting metabolic changes reflect an imbalance in liver energy metabolism, due to excessive delivery of lipids and other substrates. Excess nutrients overwhelm the liver’s capacity to oxidize these substrates or export them after incorporation into very-low-density lipoproteins (VLDLs). The major metabolic and pathophysiological determinants leading to MASLD can be summarized as follows: (i) Increased consumption of foods containing high levels of lipids, particularly saturated fatty acids (FAs), and/or carbohydrates such as mono- and disaccharides, particularly fructose and sucrose, which in turn can activate de novo lipogenesis (DNL). (ii) The development of insulin resistance (IR) in expanded and inflamed white adipose tissue (WAT), resulting in increased lipolysis and increased fatty acid delivery to the liver. (iii) The development of hepatic IR, which is strictly related to peripheral IR and becomes more severe during disease progression. This results in insufficient suppression of hepatic gluconeogenesis, decreased glycogen synthesis and the redirection of glucose into lipogenic pathways. (iv) Development of lipid deposition in skeletal muscle, with the consequent development of local IR [3,6,10,15].

The following interconnected mechanisms represent the major contributors to the development of steatosis: (i) Increased DNL, resulting mainly from the increased delivery of glucose and other carbohydrates such as fructose to the liver and closely associated with hyperinsulinemia. This, in turn, leads to (ii) the up-regulation of key genes encoding for lipogenic enzymes that regulate DNL, mainly through the involvement of sterol regulatory element binding-protein 1c (SREBP1c), carbohydrate response element binding-protein (ChREBP), LXR or peroxisome proliferator-activated receptor γ (PPARγ). (iii) Increased delivery of FA to the liver results in an increased esterification of fatty acids into TG, involving increased diacylglycerol (DG) formation and enhanced activity of diacylglycerol O-acyltransferase 2 (DGAT2) [10,15,16].

In response to the increased FA load and TG formation, hepatocytes face several metabolic limitations: (i) decreased intrahepatic lipolysis, (ii) decreased or insufficient TG export via VLDL, and (iii) insufficient FA metabolism through mitochondrial and/or peroxisomal oxidation [10,15,16]. Some of these events can be exacerbated by variants of genes involved in lipid metabolism. In addition, increased mitochondrial β-oxidation of free fatty acids (FFAs) may be potentially deleterious. Coenzyme A-linked FFAs are shuttled into the mitochondrial matrix through carnitine O-palmitoyltransferase 1 (CPT)1 and CPT2, but this leads to excessive amounts of acetyl-CoA entering the tricarboxylic acid cycle and of NADH entering the mitochondrial electron transport chain. The resulting increase in mitochondrial oxidative metabolism can lead to an exhaustion of superoxide dismutase (SOD)-2 and glutathioneperoxidase activity, ultimately resulting in the increased production of reactive oxygen species (ROS) and of oxidative stress [10,16].

Disruption of lipid metabolism (as well as of glucose metabolism) represents a key driver not only of MASLD progression but also of MASH-related HCC development [11]. Indeed, the interconnected metabolic abnormalities described above (IR, increased DNL and gluconeogenesis, increased hepatic delivery and synthesis of FA, impaired β-oxidation, increased oxidative stress, and persistent inflammatory responses) act synergistically to promote hepatic fat accumulation, inflammation, and fibrosis, thereby creating a pro-carcinogenic microenvironment. For example, abnormal glucose and lipid metabolism can promote HCC development by stimulating cell proliferation, inhibiting apoptosis and inducing genetic mutations [11].

An additional relevant role in MASLD progression is played by LXR/cholesterol pathways. LXRs are members of the nuclear receptor superfamily and pivotal regulators of lipid metabolism [17]. Elevated intracellular cholesterol concentrations trigger the synthesis of oxysterols, which in turn activate LXR-dependent transcriptional programs. These programs facilitate cholesterol efflux, suppress de novo synthesis and influx, and coordinate the metabolism of phospholipids and FA [18,19]. LXRs play a central, yet paradoxical, role in MASLD and MASH [16]. Patients with MASLD/MASH exhibit significantly increased hepatic expression of LXRα and its target genes, which correlated with the severity of steatosis, inflammation, and fibrosis [16]. Although LXR activation may be beneficial in reducing hepatic cholesterol levels and inflammation, it simultaneously promotes FA accumulation by increasing DNL, thereby driving and worsening liver steatosis. Studies in mice lacking LXR and fed lipid-enriched diets have reported resistance to obesity, decreased lipogenic gene expression and reduced steatosis; however, these mice still accumulate cholesterol that promotes steatohepatitis [16].

## 2. The Drivers of MASLD Progression to MASH, Liver Fibrosis, Cirrhosis and HCC

The subject analyzed in this section would in itself require a dedicated review, but since the present review is dedicated to the role of the OSM/OSMRβ axis, only a selection of major issues will be briefly proposed here as a general guide to the current understanding of how MASLD can progress. Interested readers can find more detailed descriptions of the issues discussed in a number of excellent and authoritative reviews [3,6,10,11,15,16]. According to the current literature, MASLD progression is a very complex process involving several mediators, cell types and mechanisms involving critical events, such as hepatocyte injury and death, chronic inflammatory response, fibrogenesis, genetic variants, lipotoxicity, oxidative stress and many others, including gut microbiome and dysbiosis. As previously mentioned, simple liver steatosis is detected in 75–80% of patients with a diagnosis of MASLD, usually in obese and/or T2D patients. Although just a subset of patients with simple steatosis may progress to MASH and advanced fibrosis, these patients remain at significant risk to develop cardiovascular diseases and extrahepatic cancers [3,10,11,15,16].

### 2.1. Hepatocyte Injury and Death: A Matter of Altered Metabolism, Lipotoxicity, Oxidative Stress and Mitochondrial Dysfunction

From a histopathological perspective, MASH is characterized by features of sublethal hepatocyte injury (often referred to as *ballooning degeneration*) [20] as well as of evidence of cell death. According to the current literature, cell death can be ascribed to different forms of *regulated cell death*, mainly including apoptosis, necroptosis, pyroptosis and ferroptosis, as extensively reviewed elsewhere [21,22,23,24]. Although the relative quantitative contribution of these different types of cell death to disease progression remains unclear, damage-associated molecular patterns (DAMPs) released by dead hepatocytes are undoubtedly critical to the chronic induction of inflammatory response under MASH conditions [10,15,21,22,23,24].

Hepatocyte injury and death under MASH conditions can be triggered by different stimuli, factors and conditions but with lipotoxicity, oxidative stress and mitochondrial dysfunction playing the most relevant roles [10,15,21,22,23,24]. Lipotoxicity deserves a brief mention here since it is a consequence of the previously described excess hepatic delivery of lipid substrates to as well as the associated impairment of lipid-related metabolic pathways. This particular scenario is believed to generate lipotoxic lipids that, by inducing critical events such as mitochondrial dysfunction, endoplasmic reticulum (ER) stress and oxidative stress, ultimately trigger hepatocyte injury and death and then genomic instability, persistent inflammation, and chronic activation of fibrogenesis [10,15,21,22,25]. Monounsaturated FA (particularly palmitate and stearate), ceramides, lysophosphatidyl choline, lysophosphatidic acid and diacylglycerols are the major directly cytotoxic lipids. These toxic lipids have been reported to induce apoptosis through the activation of c-Jun-N-terminal kinases (JNK) and mitochondrial death pathways, as well as through induction of ER stress and inflammasome activation [22,25]. In addition, lipotoxicity can induce the release of extracellular vesicles (EVs) from fat-laden hepatocytes, which can exert pro-inflammatory, pro-fibrogenic and pro-angiogenic effects on surrounding cells [26,27,28,29]. Cell injury can be exacerbated by free cholesterol [16,17] and its accumulation in hepatocytes, Kupffer cells (KCs) and hepatic stellate cells (HSCs), which results in oxidative stress, mitochondrial dysfunction and ATP depletion, leading to either apoptosis or necrotic-like cell death [10,22].

### 2.2. Genetic Variants

Apart from the mechanisms that can lead to hepatocyte injury and death, it now clear that MASLD progression to more advanced stages of the disease can occur primarily in patients carrying one or more genetic variants in genes encoding for proteins involved in lipid metabolism (patatin-like phospholipase domain containing 3 or PNPLA3, transmembrane 6 superfamily member 2 or TM6SF2, membrane bound O-acyltransferase domain-containing 7 or MBOAT7), glucose uptake (glucokinase regulator or GCKR), VLDL secretion (apolipoprotein B or APOB, microsomal triglycerides transfer protein or MTTP), mitochondrial lipid metabolism (uncoupling protein 2) or in the control of oxidative stress, inflammation and fibrogenesis (collagen-type XIII alpha 1 chain or COL13A1, EF-Hand Calcium Binding Domain 4B or EFCAB4B, Farnesyl-diphosphate farnesyltransferase 1 or FDFT1, Interferon Lambda 4 or IFNL4) [22,30,31,32]. These gene variants have been reported to be strongly associated not only with susceptibility to the development of MASLD but also with susceptibility to MASH and fibrosis (such as PNPLA3, TM6SF2, APOB, MBOAT7, IFNL4) and even to the development of HCC (PNPLA3 and APOB). This means that in each individual, the presence of these variants represents an additive and critical driver of progression in addition to the metabolic and dietary ones. Moreover, it has been proposed that MASLD itself should be considered a heritable disease on the basis of epidemiological, familial aggregation and twin studies [30,31,32]. Interested readers can find more extensive and detailed analyses of the relationships between the presence of genetic variants, the susceptibility to MASLD and disease progression in a number of authoritative reviews [31,32,33,34].

### 2.3. Chronic Inflammatory Response: A Focus on the Role of Macrophages in MASLD Progression

MASLD progression within the previously described scenario of dysregulated metabolism and persistent hepatocyte injury and death is driven by two essential and interconnected processes: the chronic inflammatory response and the chronic activation of repair mechanisms (i.e., liver fibrogenesis, see the next section) [10,15,24,35].

**Figure 1:**
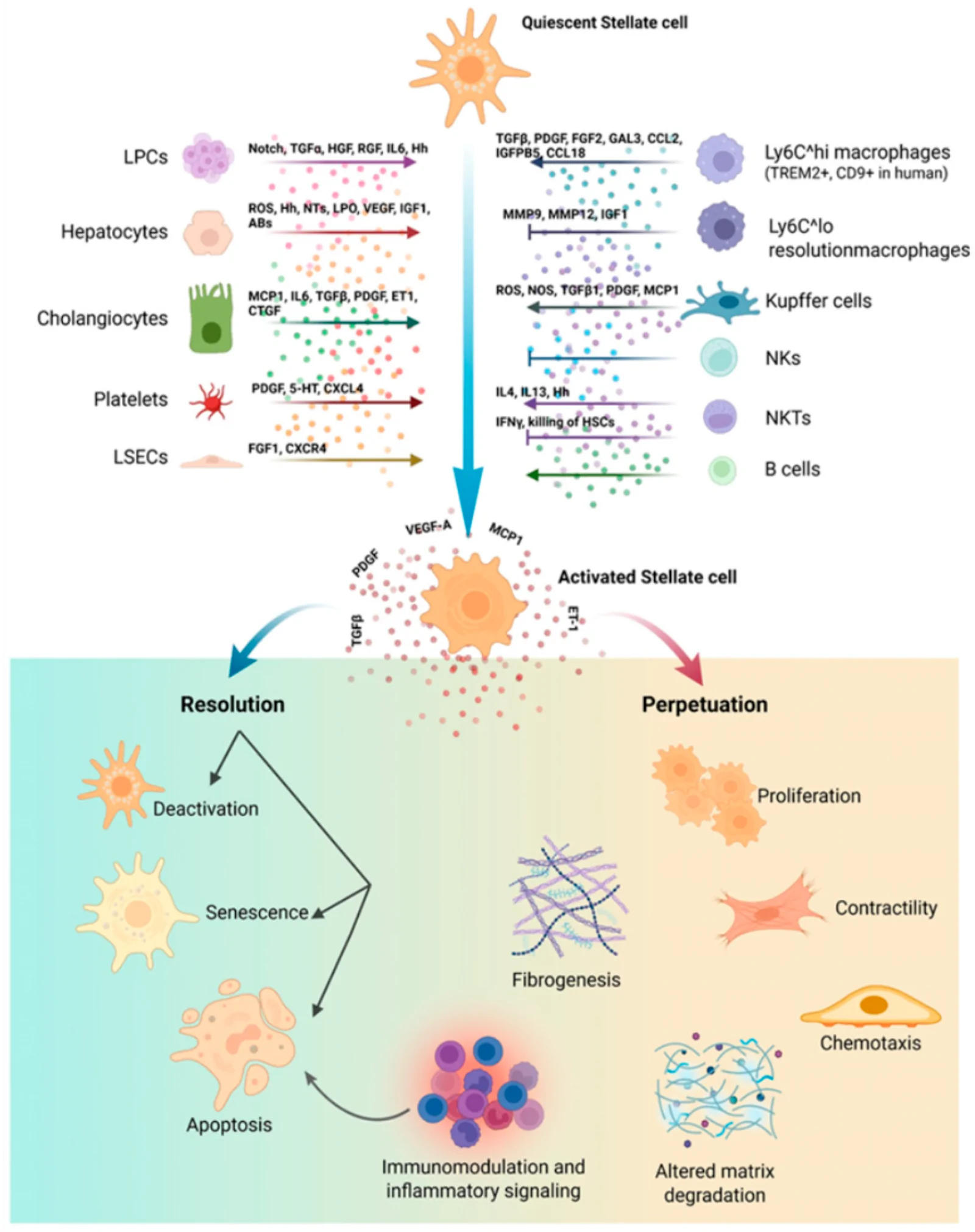
Cell-to-cell signaling network regulating hepatic stellate cell (HSC) activation, resolution and perpetuation of the fibrogenic response. Quiescent HSCs are exposed to signals from parenchymal and non-parenchymal liver cells: liver progenitor cells (LPCs, via Notch, TGFα, HGF, RGF, IL6, Hedgehog (Hh)), hepatocytes (ROS, Hh, nucleotides (NTs), lipid peroxidation (LPO) products, VEGF, IGF1, apoptotic bodies (ABs)), cholangiocytes (MCP1, IL6, TGFβ, PDGF, ET1, CTGF), platelets (PDGF, serotonin (5-HT), CXCL4) and liver sinusoidal endothelial cells (LSECs, via FGF1, CXCR4), together with signals from innate and adaptive immune cells like Ly6C^high and Ly6C^low macrophages (TREM2+, CD9+ in humans), Kupffer cells, NK cells, NKT cells and B cells, releasing TGFβ, PDGF, FGF2, GAL3, CCL2, IGFBP5, CCL18, MMP9, MMP12, IGF1, ROS, NOS, TGFβ1, MCP1, IL4, IL13, Hh and IFNγ. Upon activation, HSCs release VEGF-A, PDGF, TGFβ, MCP1 and ET-1 (acting both in a autocrine and paracrine manner), driving either Resolution (through deactivation, senescence and/or apoptosis) or Perpetuation of the fibrogenic response (characterized by HSC proliferation, contractility, chemotaxis, fibrogenesis, altered matrix degradation and immunomodulatory/inflammatory signaling). Created in BioRender. Cannito, S. (2026) https://BioRender.com/ky060fz.

The persistent inflammatory response fuels progression and fibrogenesis and involves primarily several innate and adaptive immune cells. The application of novel single-cell multiomic technologies as well as of spatial trancriptomics, imaging mass cytometry and other advanced techniques has started to reveal several major features: (i) the hepatic landscape of innate and adaptive immune responses in either patients or murine models is significantly reshaped during steatohepatitis, a process that deeply affects disease progression and HCC development [36,37,38,39]; (ii) MASLD-associated inflammation reflects systemic changes and is sustained and/or modulated by multiple intrahepatic and extrahepatic factors, with inflammatory signals originating from adipose tissue, the gut, skeletal muscle, and bone marrow; (iii) the disease progression relies on an extremely complex network of cell-to-cell interactions among innate immunity cells such as KC, monocyte/macrophages and neutrophils recruited from peripheral blood, and dendritic cells; adaptive immune cells, including B and T lymphocytes, cluster designation (CD) 4+ and CD8+ T lymphocytes, and T_H_17 cells; innate lymphoid cells like NK and NKT cells, regulatory T cells (Treg); hepatic myofibroblasts and their precursor cells (mainly HSC and portal fibroblasts), as well as injured hepatocytes, activated platelets and sinusoidal endothelial cells (SECs). This very complex scenario is briefly summarized in Figure 1 [10,15,22,24,25,35,36,37,38,39]. In this section, we will primarily focus on the prominent role of macrophages, but interested readers can find more details about the role of other innate or adaptive immunity cells in MASLD progression in a number of recent and authoritative reviews [10,15,36,37,40]. The role of adaptive immunity will be revisited later in relation to the development of MASH-related HCC.

**Figure 2:**
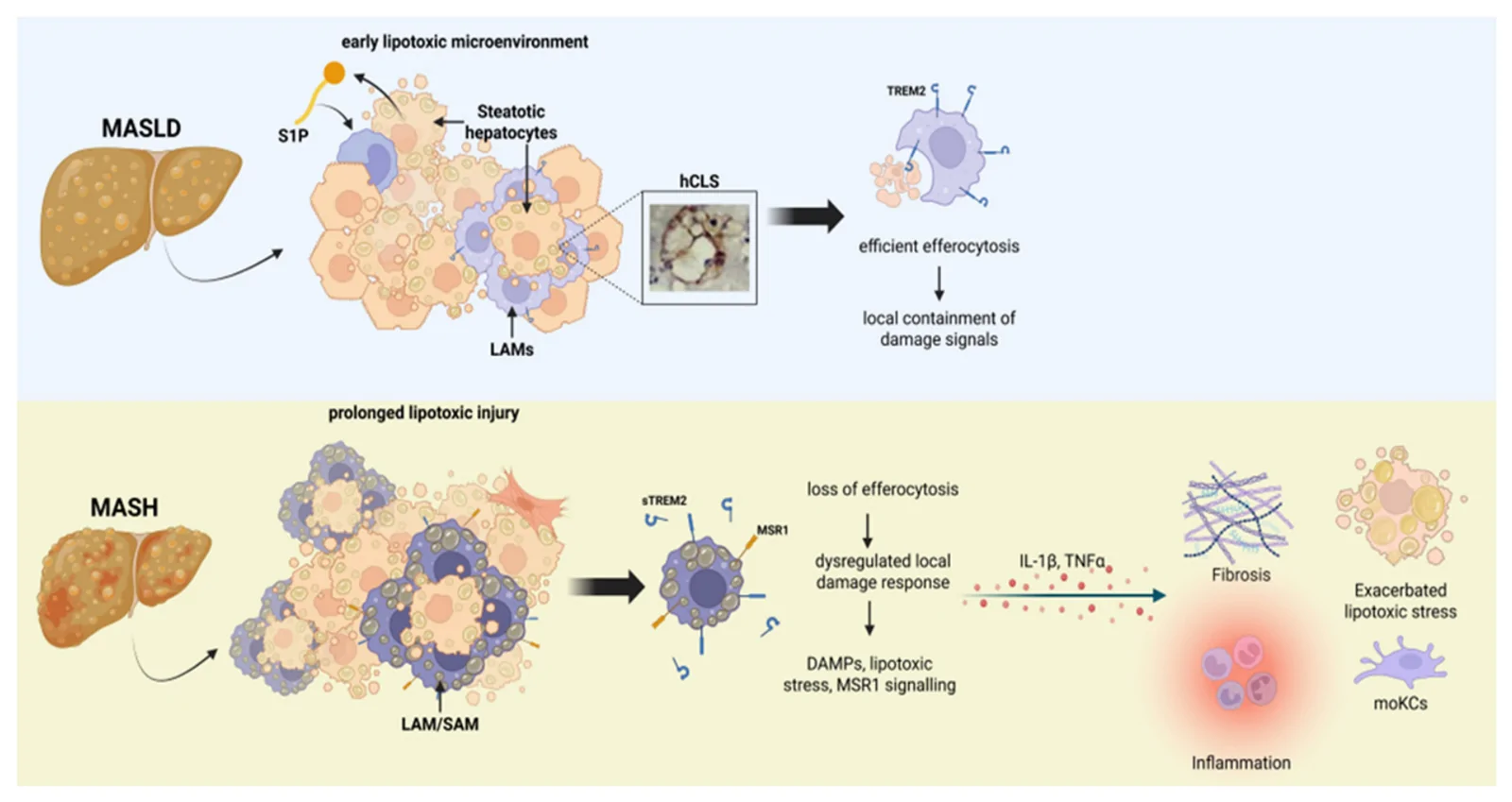
Efferocytosis capacity distinguishes early MASLD from established MASH. In early MASLD, steatotic hepatocytes release sphingosine-1-phosphate (S1P), promoting the formation of hepatic crown-like structures (hCLS), in which lipid-associated macrophages (LAMs) expressing TREM2 (Triggering Receptor Expressed on Myeloid cells 2) efficiently clear damaged/dying hepatocytes (efferocytosis), locally containing damage-associated signals. Upon prolonged lipotoxic injury in MASH, this protective mechanism is lost: macrophages showing a LAM/scar-associated macrophage (SAM) phenotype release soluble TREM2 (sTREM2) and engage MSR1 (Macrophage Scavenger Receptor 1) signaling, resulting in a dysregulated local damage response with accumulation of damage-associated molecular patterns (DAMPs) and persistent lipotoxic stress. The consequent release of IL1β and TNFα sustains fibrosis, exacerbated lipotoxic stress and expansion of monocyte-derived Kupffer cells (moKCs), fueling chronic inflammation. Created in BioRender. Cannito, S. (2026) https://BioRender.com/nhturqw.

In this scenario, a major role is unequivocally played by the activation of KCs, which is particularly relevant in the early phases of the disease, and by that of macrophages recruited from circulating monocytes (MoMs) becoming predominant afterwards [36,37,41]. Literature data indicate that macrophages play a dual role in the initiation and progression of MASLD and MASH. The following major issues regarding MASLD progression can be highlighted: (i) Under physiological conditions, KCs perform several functions as resident macrophages and represent a heterogeneous cell population in both the human and murine liver [41]. During the early stages of MASLD, they acquire a pro-inflammatory phenotype through the activation of pattern recognition receptors (PRRs) by DAMPs (ADP, free cholesterol, mitochondrial DNA, etc.) released by injured and fat-laden hepatocytes as well as by pathogen-associated molecular patterns (PAMPs) reaching the liver as a consequence of intestinal barrier dysfunction and microbial dysbiosis. (ii) Under conditions of persisting injury, KCs diminish in number, and the inflammatory response is then sustained by macrophages derived from infiltrated Ly6C^high^ monocytes (MoMs), which replenish the KC niche and form the so-called TREM2+ lipid-associated macrophages (LAMs) [41,42]. (iii) TREM2+ LAMs form the so-called hepatic crown-like structures (hCLS), which are histopathologically identifiable structures in which macrophages accumulate around fat-laden and degenerating hepatocytes. During this early phase, TREM2+ LAMs have an effective efferocytic capacity that enables them to clear apoptotic cells and limit the detrimental accumulation of free lipids, thereby reducing the release of DAMPs and the inflammatory response. (iv) Under conditions of persistent lipotoxic injury, LAMs shed TREM2 (which is released as soluble TREM2) and progressively lose their efferocytic capacity. LAMs or TREM2+ macrophages are subsequently transformed into fully activated pro-inflammatory cells releasing IL1β, TNFα and other mediators (see Figure 2) that drive both inflammation and fibrogenesis.

Before concluding this section, we would like to mention the potential role of the “liver-spleen axis” in the development of CLD, which is defined as a bidirectional interaction between the spleen and the liver through vascular, immunological and metabolic pathways [43]. The concept of the liver–spleen axis is supported by clinical observations demonstrating a close relationship between the two organs during CLD progression. Splenomegaly and hypersplenism are well-recognized manifestations of liver cirrhosis [44], whereas in MASLD patients, spleen enlargement has been reported to correlate with the severity of hepatic fat accumulation, further suggesting the existence of functional crosstalk between the liver and the spleen [45,46]. Since, to the best of our knowledge, no evidence currently directly links OSM or OSM-related pathways directly to the liver–spleen axis in MASLD, we will only briefly summarize some of the major concepts emerging from recent studies, particularly those related to inflammatory and immune mechanisms [43,47,48]. As is well known, the spleen serves as a major immunological organ, as well as a reservoir and modulator of myeloid and lymphoid cells. Recent evidence suggests that obesity and MASLD are associated with a splenic immune remodeling that may contribute to the maintenance of systemic low-grade inflammation and the amplification of hepatic inflammatory responses [43,49]. In particular, we would like to focus on the following points: (i) the liver–spleen axis has been proposed to sustain obesity-induced systemic inflammation and fatty liver disease through the enrichment and activation of myeloid-derived suppressor cells (MDSCs) and NK/NKT cell populations, which subsequently modulate hepatic immune responses [50]; (ii) experimental evidence suggests that CD11b+ CD43^hi^ Ly6C^lo^ splenocyte-derived macrophages may contribute to the expansion of the hepatic macrophage compartment during MASLD progression to promote chronic inflammation and fibrogenesis [51]; (iii) a very recent study, which confirmed the presence of enlarged spleens in patients with MASLD, identified induced TRNP1hi CD8+ T cells in the spleens of MASLD/MASH mouse models and patients. These cells exhibited pro-fibrogenic properties through the secretion of INSR-α that ultimately contribute to activating HSC [52]. Overall, although the potential interaction between the OSM/OSMRβ axis and the liver–spleen axis has not yet been investigated, these studies suggest that the spleen may act as an active contributor to liver disease progression, an issue that deserves further investigation. Interested readers may find more information about the role of the spleen–liver axis in a recently published review [43].

### 2.4. Fibrogenesis

Liver fibrogenesis, the biological process leading to fibrosis (i.e., the excessive deposition of extracellular matrix or ECM), is mainly sustained by a heterogeneous population of hepatic α-smooth muscle actin-positive (αSMA) cells defined as myofibroblasts (MFs). MFs originate from different precursor cells of mesenchymal origin through a process defined as of activation/transdifferentiation [10,53,54,55,56]. Hepatic MFs, in addition to being activated by a plethora of mediators (growth factors, cytokines, chemokines, ROS, adipokines, proangiogenic factors, released by KC and macrophages, damaged hepatocytes, SEC, platelets, cholangiocytes, NK cells, NKT cells and other cells of the adaptive immunity (i.e., as in any form of CLD)), can also respond to metabolic signals as well as signals related to gut dysbiosis or delivered from adipose tissue [10,53,54,55,56]. The following major issues regarding the role of liver MFs in the MASLD/MASH (see also Figure 1) can be summarized as follows [10,53,54,55]: (i) In progressive MASLD, MFs originate mainly through the activation/transdifferentiation of quiescent HSC and only marginally from other precursors like portal fibroblasts. (ii) As described for other CLD of different etiologies, MFs exhibit classical phenotypic responses, including (a) active proliferation in response mainly to platelet-derived growth factor (PDGF), transforming growth factor α (TGFα), connective tissue growth factor (CTGF), basic fibroblast growth factor (bFGF) and leptin; (b) excess deposition of ECM as induced by transforming growth factor β1 (TGFβ1), bFGF, CTGF); (c) reprogramming of ECM remodeling due to the overexpression of tissue inhibitors of metalloproteases (TIMP) and of matrix metalloproteases (MMPs) that are unable to efficiently remove fibrillar collagen; (d) a pro-angiogenic role responding to hypoxic conditions through the release of vascular endothelial growth factor (VEGF), Angiopoietin-1 and -2, Hedgehog ligands; (e) a pro-inflammatory role involving the release of CC chemokine ligand-2 or -21 (CCL2, CCL21), interleukin 1β (IL1β), responses to several inflammatory mediators and the ability to migrate (as induced mainly by PDGF, CCL2, VEGF, Angiotensin I). (iii) The removal of the etiological cause of CLD is the predominant event that allows fibrosis to arrest or even regress. Fibrosis regression is accompanied by a number of relevant changes, including the decreased synthesis of pro-inflammatory and pro-fibrogenic mediators, the decrease in the synthesis of ECM components and the predominance of MMP activity over TIMP activity. When this ideal condition occurs, approximately 50% of HSC-derived MFs can undergo apoptosis, an event reported to be induced by NKT and pro-resolution macrophages. HSC-derived MFs that escape apoptosis can either become inactivated or dedifferentiated or become senescent and subsequently undergo apoptosis, possibly with the active contribution of activated NK cells.

It should be noted that recent data obtained using single-cell transcriptomics have revealed that HSCs should be considered a more heterogeneous cell population than previously believed [57]. In addition, HSCs have been proposed to contribute to the regulation of carbohydrate, mitochondrial and lipid homeostasis, suggesting that in order to undergo activation/transdifferentiation into MFs, HSCs need to rapidly adapt metabolically [56]. Accordingly, these adaptations should include the reprogramming of carbon metabolism and enhanced mitochondrial number and activity, as well as ER stress, and the release of FFA through the autophagy-dependent hydrolysis of retinyl esters stored in cytoplasmic droplets [56].

The available literature clearly indicates that HSCs as well as HSC-derived MFs during the course of any CLD, including progressive MASLD, are able to establish extensive crosstalk with practically all other cell types involved in disease progression. This means that HSCs and MFs are cells capable of sensing a plethora of signals and mediators and to respond to them by releasing, in an autocrine/paracrine manner, several mediators, as unequivocally shown for TGFβ1, PDGF, VEGF, CCL2 and other mediators [10,53,54,55,56,58]. This scenario is summarized in Figure 1.

It should be emphasized that all these cell-to-cell interactions are critical not only for MASLD progression but also for HCC development, with several ligand–receptor axes indicated as putative biomarkers of progression or suitable molecular targets for novel therapeutic strategies [3,6,7,11,15,35,37,40]. In the next sections of this review, we will focus on the emerging role of the OSM/OSMRβ axis in CLD progression and HCC development, with a focus on MASLD and MASH.

## 3. Oncostatin M: Introducing a Peculiar and Enigmatic Cytokine

### 3.1. Oncostatin M: Introductory Remarks

Oncostatin M (OSM) is a 22–26 kDa pleiotropic and multifunctional cytokine (depending on the species) belonging to the interleukin-6 (IL6) family of cytokines [59] that was originally identified as a protein produced by U937 cells (a cell line derived from human histiocytic lymphoma) [60]. The IL6 cytokine family also includes leukemia inhibitory factor (LIF), a number of interleukins (IL11, IL27, IL30 and IL31) cardiotrophin-1, ciliary neurotrophic factor and cardiotrophin-like cytokine factor 1 [61,62,63,64]. In particular, OSM shares a high level of homology (structural, functional and genetic) with LIF [59,62], possibly due to an ancestral event of gene duplication [65]. The name “Oncostatin M” reflects the initial finding that identified this cytokine as cytostatic toward melanoma cells [60]. However, literature data indicate that OSM can modulate several processes, under both physiological or pathological conditions, including cell proliferation, extracellular matrix remodeling, hematopoiesis, differentiation, inflammatory responses, angiogenesis and the acquisition of cancer stem cell markers and of a metastatic phenotype [61,62,63,64].

OSM is produced and released by different types of cells, including cells of the innate (mainly monocytes/macrophages, dendritic cells and neutrophils) and adaptive (T lymphocytes) immune system as well as hematopoietic cells, mesenchymal cells [61,62,63,64,66] and even cancer cells, as recently shown in liver cancer cells from MASH-related HCC [67].

### 3.2. OSM: Transcription, Biosynthesis and Secretion

Over the past two decades, different laboratories have cloned the human, murine and rat OSM orthologues, as reviewed in ref. [66]. Interestingly, the exon–intron structure of the OSM-encoding gene has been reported to be identical among these species, consisting of three exons separated by two introns. Moreover, the gene is always located in direct proximity to the gene encoding LIF, suggesting that both genes may have originated from a gene duplication event [68,69]. The promoters of the human (hOSM) and murine (mOSM) genes have been characterized, and available evidence indicates that they are controlled by CCAAT-enhancer-binding proteins (C/EBP) and GC-rich elements that regulate basal activity. However, signal transducer and activator of transcription (STAT)5 responsive elements are believed to be critical for stimuli-induced OSM transcription, whereas transcriptional silencer(s) (located between nt 194 and 109) in the human OSM promoter appear to ensure cell-specific control of gene expression [70,71]. For example, in hematopoietic cells, OSM transcription is strongly up-regulated by cytokines operating through STAT5, like IL2, IL6, erythropoietin and granulocyte–monocyte colony stimulating factor. OSM transcription has been reported to also be up-regulated by prostaglandin E2 via the cAMP signaling pathway [72,73]. Of interest, OSM is regarded as an amplifier of cytokine production/release [66], and both the human and murine OSM mRNAs contain several AU-rich sequences known to contribute to increased mRNA stability for several cytokines, including IL6 [74].

Following translation, full-length OSM proteins have been reported to contain from 239 to 263 amino acids with a 3D structure folded into a long-chain four-helix bundle with an up-up-down-down topology closely similar to those of other IL6 family cytokines [66]. OSM proteins are then post-translationally modified by N-glycosylation that seems to differ depending on the specific cell type [75]. Regarding hOSM (with the corresponding murine and rat data being at present less clear), following the post-translational cleavage of C-terminal prodomain and the N-terminal signal peptide, the protein results in 195 amino acids and 22 kDa, which has been reported to elicit the highest biological activity, although the 24 kDa isoform also appears to exhibit similar activity in competition assays [75]. Both protein forms can bind to the receptor complex (see the next section) with high affinity but exhibit different growth inhibitory activities: the 24 kDa isoform is 5- to 60-fold less active in growth inhibition assays using A375 melanoma cells [75].

Once secreted, OSM has been reported to bind, in its bioactive form, to various ECM components including different types of collagen (I, III, IV, VI and XI) as well as to laminin and fibronectin [76,77]. This is a unique property of OSM since other cytokines belonging to the IL6 family do not bind to ECM components. It has been proposed that OSM, within an inflammatory microenvironment, may act as a strong inducer of ECM deposition [78,79].

### 3.3. OSM, Related Receptors and OSM-Dependent Signaling Pathways

OSM acts by binding to two different heterodimeric receptors, which have in common the glycoprotein 130 (gp130), a membrane receptor protein shared by all members of the IL6 family of cytokines. OSM has been reported to first interact with the extracellular cytokine-binding domain of gp130, subsequently recruiting either the LIF receptor β (LIFRβ) to form the OSM type I receptor or to the OSMRβ to form the OSM type II receptor. The two heterodimeric receptors are differentially engaged in humans and mice: human OSM can bind to either gp130/OSMRβ or gp130/LIFRβ, whereas murine OSM binds only to the type II receptor [80,81,82,83]. Interestingly, the OSMRβ/LIFRβ ratio and the differential expression of these receptors in various cell type have been proposed as the mechanism by which OSM and LIF may exert common biological effects on particular tissues but distinct and relatively unique in other tissues [81]. It should be noted that OSMRβ is believed to mediate most OSM effects [61] by mainly activating the Janus kinase (JAK)/STAT) pathway [61], as well as the Ras–mitogen-activated protein kinase (MAPK), extracellular signal-regulated kinases (ERK) 1/2 and p38 pathways [82,83].

Regarding activation of the JAK/STAT pathway, the interaction between OSM and gp130 is known to initially induce receptor heterodimerization and then to the recruitment and activation of JAK1, JAK2 and tyrosine kinase 2 (TYK2). In particular, the C-terminal domains of both OSM receptor type I and II contain tyrosine motifs, which, once phosphorylated by JAK1 and JAK2, act as docking sites for STAT1 and STAT3. OSM has been reported to activate STAT5 and STAT6 [61,62,81].

In addition to activating the JAK/STAT pathways, OSM can also recruit the adaptor Src homology and collagen (Shc) protein to the conserved Tyr861 residue of the OSMRβ. This can lead to the activation of a number of downstream kinases such as ERK1/2, p38, or JNKs [61,66,81,83], as well as, in some cells, the phosphatidyl inositol 3-kinase (PI3K)/Akt pathway [84] and the protein kinase C δ [85]. Finally, it has been reported that OSM can also negatively modulate mitogen-activated protein MAPK signaling cascades through the recruitment of the tyrosine phosphatase SHP-2 (specifically to the Tyr759 and Tyr974 residues of the type I receptor), the recruitment of suppressors of cytokine signaling (SOCS, in particular SOCS3) on Tyr759, or through the direct action of JAK1/2 [61,66,81].

## 4. Biological Activities of Oncostatin M: A Complex Scenario

As mentioned in the previous section, OSM should be regarded as a pleiotropic and somewhat enigmatic cytokine that is produced and released by several cell types. OSM has been reported to induce or modulate various biological processes, particularly under pathological conditions. According to the current literature on pathological conditions, OSM can affect inflammatory responses, cell proliferation, angiogenesis, epithelial to mesenchymal transition (EMT), invasiveness, metastasis and the behavior of cancer stem cells (CSC) [61,62,63,64]. More recently, OSM has been reported to have a role in reshaping the tumor immune microenvironment [86].

### 4.1. OSM in Inflammatory Diseases

OSM is known to play a major role in inflammation, and it has been reported to be involved in both the acute-phase response and chronic inflammatory conditions, which may eventually result in tissue or organ fibrosis and cancer [61,62,63,64,87,88]. As already mentioned, OSM can be synthesized and released by several immune cell types in response to a variety of soluble mediators. OSM can exert either pro-inflammatory or anti-inflammatory effects according to the cellular context and the intracellular signaling elicited. OSM can act as a pro-inflammatory cytokine by stimulating both receptors (predominantly OSMRβ or type II receptor) on target cells, thereby inducing the release of several cytokines and chemokines (particularly CCL2, CCL5, CCL20 and CXC chemokine ligand [CXCL] 3), with the latter contributing to the recruitment of neutrophils and monocytes [61,62,63,64,87,88]. Interestingly, the anti-inflammatory action of OSM is mediated primarily through the type I receptor (LIFR). Moreover, under chronic inflammatory conditions, OSM and OSMRβ are often overexpressed, with OSM generally regarded as a cytokine capable of sustaining inflammation and to promoting fibrosis.

In particular, the role of OSM has been investigated in several chronic inflammatory diseases, including lung diseases, inflammatory bowel disease, cardiovascular diseases, skin diseases, arthritis, COVID-19 and many other conditions, as reviewed in ref. [64]. It should be noted at the outset that OSM has been reported to exert either positive or negative effects on different diseases, with strategies designed to inhibit OSM-related signaling being beneficial in some diseases but detrimental in others [64]. Here, we will briefly focus only on a few relevant examples of diseases in which OSM and OSM-related signaling have been proposed to be relevant. Interested readers can refer to a more comprehensive review [64].

Regarding lung diseases, for example, OSM has been reported to be up-regulated and to play a critical role in patients affected by pulmonary fibrosis and asthma. In both diseases, as has also been commonly observed in diseases affecting other tissues, OSM up-regulation paralleled the extent of lung chronic inflammation, the accumulation of profibrotic macrophages and the excessive deposition (particularly in lung fibrosis) of ECM components, suggesting that OSM and related pathways may sustain the development and progression of these lung diseases [89,90,91].

Another interesting example is represented by inflammatory bowel diseases (IBD) such as Crohn’s disease and ulcerative colitis, in which OSM and OSMR are overexpressed in many IBD lesions [92]. Moreover, a retrospective study showed that patients with increased levels of OSM and who were treated with anti-TNFα therapies had, one year later, a lower chance of remaining in remission [93]. Similarly, IBD patients having high levels of OSM and OSMR expression before infliximab therapy showed a significantly lower response to treatment [94]. In the latter study, the use of OSM-knockout mice in a murine IBD experimental model resulted in a highly significant decrease in disease severity, confirming that OSM signaling not only represents a key feature of IBD pathogenesis but can also negatively interfere with anti-TNFα therapies [95]. Along these lines, it is interesting to note that JAK/STAT inhibitors like tofacinib (inhibitor of JAK1 and JAK3) and filgotinib (selective inhibitor of JAK1) are currently being evaluated in clinical trials for IBD, as reviewed in ref. [64].

A closely related scenario has emerged from studies on rheumatoid arthritis, in which OSM and IL1β once again play critical roles in disease pathogenesis and progression [64]. In vitro studies have shown that OSM increases the deleterious effects of TNFα through the activation of STAT3 signaling and that tofacinib exerts significant positive effects [94,95]. In an in vivo study using a murine model of arthritis, the administration of anti-OSM antibodies resulted in a significant improvement in the experimental disease [96], leading to the design of dedicated clinical trials employing anti-OSM therapeutics (GSK315234 and GSK2330811) [64].

Unlike its role in IBD and arthritis, the role of OSM in cardiovascular disease remains controversial. Persistent OSM-dependent STAT3 activation has been reported by some studies to exert detrimental effects on arterial vessels and to promote atherogenesis [97,98]. However, another study indicated that chronic OSM administration in mice reduced atherosclerosis development; moreover, patients with higher serum levels of OSM showed improved survival to coronary heart disease [99]. Experimental in vivo studies have also indicated that OSM increases cardiac function following myocardial infarction [100,101].

### 4.2. OSM in Cancer Biology

The multifaceted role of OSM is emphasized by studies investigating its involvement in cancer biology. There is general agreement that OSM (and/or OSM/OSMRβ axis) plays a predominantly deleterious role in cancers affecting several different tissues and organs. The available literature on solid human cancers (see, for a detailed review, ref. [64]) indicates that the involvement of OSM is sometimes particularly relevant in specific cancer types, including, for example, ductal carcinoma of the breast, ductal adenocarcinoma of the prostate and of pancreas, adenocarcinoma of the lung and of colon, squamous cell carcinoma of the cervix and of the skin. With regard to brain cancers, OSM has been implicated in practically all major types (i.e., astrocytoma, astroglioma, glioblastoma, medullo blastoma, etc). A detailed analysis of the involvement of OSM in human cancer is beyond the scope of this review, and we will, therefore, highlight only some of the most common findings reported in refs. [62,63,64]: (i) in most human solid tumors, OSM is usually overexpressed, particularly in the tumor mass, and the expression has been reported for some tumors to correlate with poor prognosis; (ii) in several tumors, it has been outlined that OSM and the OSM/OSMRβ axis (often through involvement of STAT3) are strictly related to increased epithelial-to-mesenchymal transition (EMT) and increased invasiveness and metastasis; (iii) another common finding is that activation of the OSM/OSMRβ axis results in increased pathological angiogenesis, with a direct correlation between OSM and VEGF expression; (iv) in some cancer types, although not all, OSM is able to stimulate proliferation of cancer cells; (v) OSM is emerging as a cytokine potentially capable of reshaping the tumor immune microenvironment (TIME). A few isolated examples of beneficial effects exerted by OSM have also been reported in specific cancers such as chondrosarcoma, in which OSM apparently induced cell cycle arrest and enhanced apoptosis in cancer cell lines [64,102] and melanoma, in which OSM has been reported to inhibit cancer cell proliferation [64,103].

## 5. The Role of OSM in Chronic Liver Diseases and in MASLD/MASH

According to the aforementioned literature, OSM and the related OSMRβ/JAK/STAT signaling axis have been reported to regulate critical biological processes under normal and pathological conditions, including cell proliferation, extracellular matrix remodeling, hematopoiesis, differentiation, inflammatory responses, angiogenesis, acquisition of cancer stem cell markers and of a metastatic phenotype. In the liver, these processes also include hepatic development and regeneration [59,61,63,64,87,104]. All these critical processes are known to be deeply involved in the progression of CLDs of different etiologies toward more advanced pathological stages, including cirrhosis and liver failure as well as HCC development [41,53,54,56,57]. In the following sections, we will analyze literature data on the roles of OSM in CLDs, with a focus on progressive MASLD and MASH-related HCC. For interested readers, we also note, although not reviewed in this study, that OSM is emerging as a mediator involved in the development of intrahepatic cholangiocarcinoma [63].

### 5.1. OSM in Chronic Liver Diseases: A Pro-Inflammatory and Pro-Fibrogenic Mediator

One of the earliest in vivo studies involving OSM in CLD was an immunohistochemistry study performed on a limited number of human specimens obtained from either cirrhotic livers (n = 6) and donor livers (n = 4) not used for liver transplantation [105]. This preliminary morphological study concluded that OSM was mainly expressed by CD68-positive cells, most likely Kupffer cells. Its expression was low and variable in control livers but significantly higher in cirrhotic livers. The authors of this pioneering study also suggested that the expression of OSMRβ, which was very low in normal hepatocytes, was apparently unchanged in cirrhotic livers, with LIFRβ being weakly expressed in normal livers but more intensely expressed in cholangiocytes of reactive biliary ductules in cirrhotic livers [105]. Although the authors analyzed only a few cirrhotic livers of unreported etiology, these data supported previous studies from the same research group indicating that OSM was able to up-regulate the expression of collagen type I and of TIMP1 by liver MFs derived from activated HSCs [106]. To our knowledge, these two studies were the first to identify OSM as a putative profibrogenic mediator. In those years, another study reported that knockout (ko) mice for OSMRβ treated with the hepatotoxin carbon tetrachloride (CCl_4_) or subjected to partial hepatectomy showed impaired liver regeneration compared with control mice. Moreover, reduced expression of TIMP1 and TIMP2 was also reported in these KO mice, thus suggesting that OSM is a mediator involved in hepatocyte proliferation and tissue remodeling [107]. Along these lines, one study showed that OSM, in addition to being expressed by KC, was also expressed in oval cells in the rat model of 2-acetylaminofluorene/partial hepatectomy and regeneration. Moreover, the OSM receptor was found mainly on oval cells, suggesting that the OSM/OSMR system may be critical for promoting oval cell differentiation into hepatocytes [108]. In addition, in a rat experimental model of liver injury induced by repeated injections of dimethylnitrosamine, it was shown that OSM operated as a key mediator for hepatocyte proliferation and survival [109]. A subsequent study provided the first evidence suggesting that OSM may contribute to liver angiogenesis, a critical process in both physiological and pathological conditions, by up-regulating the transcription and synthesis of hypoxia-inducible factor (HI) 1α in HepG2 cells as well as in the PH5CH8 human hepatocyte through OSM-dependent JAK/STAT3 and ERK1/2 signaling pathways. Accordingly, this HIF1α up-regulation also resulted in increased expression of HIF1-downstream genes encoding for VEGF and plasminogen activator inhibitor 1 [110]. Interest in the role of OSM in CLD was renewed by an in vitro study reporting that the administration of OSM to cultured human HepG2 cells resulted in a strong up-regulation of GP73 [111], a glycoprotein proposed as a biomarker of cirrhosis and HCC [112,113]. Interestingly, the response of HepG2 cells to OSM was far greater than their response to IL6.

The involvement of OSM in CLD progression was proposed relatively early [92,93,94], based in part on preliminary data indicating that OSM was able to up-regulate collagen type I and TIMP1 expression in activated HSC or liver MF [106,114] as well as evidence that hepatocyte proliferation and tissue remodeling were impaired following persistent liver injury in mice with genetic deletion of the OSM receptor [107]. However, the first convincing study that mechanistically correlated OSM to liver fibrogenesis was not published until 2017 [115]. In this study, OSM was proposed to act as a pro-fibrogenic mediator by regulating the activation of macrophages during chronic liver injury. The authors of this study employed mice with genetic deletion of OSM that were then chronically treated (12 weeks) with the hepatotoxin thioacetamide (TAA). OSM deletion resulted in a significant reduction in liver fibrosis and in the expression of both collagen 1a1 and TIMP1 compared with wild-type animals, without affecting parameters of hepatocyte injury. Moreover, the exposure of co-cultures of HSC and liver macrophages to recombinant OSM resulted in significantly increased expression of critical pro-fibrogenic mediators like PDGF and TGFβ together with the up-regulation of TIMP1 by HSCs. In addition, continuous OSM expression, obtained by employing the procedure of hydrodynamic tail vein injection of OSM, was sufficient to induce liver fibrosis. Finally, by overexpressing OSM and performing fluorescence-activated cell sorting, these authors proposed that macrophages recruited from peripheral blood were responsible for OSM-dependent pro-fibrogenic activation of HSC [115]. Although this study was partly performed using the TAA model of chronic liver injury that is not fully representative of the human condition, it was the first to unequivocally demonstrate the pro-fibrogenic action of OSM [115].

A study from our laboratory provided further support for the concept that OSM can operate as a pro-fibrogenic mediator by showing that human recombinant OSM (hrOSM) was able to stimulate the oriented migration (i.e., chemotaxis) of human MF-like LX2 cells, originally derived from activated HSC [116]. In particular, OSM was reported to stimulate MF migration by eliciting an increase in intracellular ROS and through activation of STA1/STAT3 (with activation of Ras/Erk, JNK1/2 and PI3/Akt signaling pathways also detected in LX2 cells). In addition, OSM also induced the recruitment and stabilization of HIF1α, confirming what was previously described by other authors [110]. This finding is relevant since, in the same study, evidence was provided that OSM-dependent migration relied on a biphasic mechanism requiring the early intracellular generation of ROS and the subsequent HIF1α-dependent expression and release of VEGF. VEGF, in turn, contributes to oriented migration of LX2 cells stimulated by OSM. hrOSM was also able to increase the transcription of pro-inflammatory mediators like the chemokine CCL2 and the cytokines IL6 and TNFα in LX2 cells [116].

OSM was also proposed in a clinical study to be an essential component of an immune gene signature capable of predicting advanced fibrosis in CLD of mixed etiologies. The study analyzed snap-frozen liver tissues from patients at different stages of fibrosis. The other components of the immune signature were all factors related to chronic injury and inflammation, including, in addition to OSM, VEGFA, Chitinase 1, Fc fragment of IgE–high-affinity I receptor for gamma polypeptide and zeta chain of T-cell receptor-associated protein kinase 70. This signature was reported to discriminate between patients with a low grade of fibrosis (i.e., F1 andF2) from those with more advanced stages of fibrosis (F3 and F4). Interestingly, the signature was reported to be independent on pathological and clinical features, with OSM and other factors reported to decrease in more advanced stages [117]. In a more recent study using a rat model of liver fibrosis induced by chronic administration of CCl_4_, OSM was found to be the main up-regulated cytokine (together with IL1β). Moreover, OSM was reported to be produced and released by KCs, macrophages and endothelial cells. Interestingly, according to this study, the target cells were hepatocytes, endothelial cells and HSCs, all of which expressed the necessary receptors [118].

A final point concerning liver fibrogenesis relates to a recent study investigating early events in a murine model of alcohol-associated liver disease (ALD), which revealed an interesting relationship between OSM and Yes-associated protein (YAP) [119]. YAP is a transcriptional coactivator and downstream effector of the Hippo signaling pathway, which has been implicated by a number of studies in the pathogenesis of various liver diseases [120,121]. YAP^−/−^ mice, mice injected with an adeno-associated virus 9-delivered saCas9/sgYAP system or YAPΔHep mice were fed the Gao-binge diet to mimic early ALD events. YAP deficiency was found to exacerbate early features of ALD, including steatosis and inflammatory response, whereas overexpression of YAP in hepatocytes significantly reversed these aspects. In particular, hepatocellular YAP was reported to be a negative regulator of OSM/STAT3-dependent up-regulation of CD36 [119], a well-known, critical membrane multifunctional protein that regulates the uptake of fatty acids in several cells, including hepatocytes, that play a key role in lipid metabolism, inflammation and immune response.

### 5.2. OSM in the Progression of MASLD/MASH

Studies investigating the involvement of OSM and related signaling pathways in MASLD/MASH (or in NAFLD/NASH, as for the previous disease nomenclature) are surprisingly few and have yielded somewhat contradictory results. Given the current definition of MASLD as a spectrum of conditions encompassing steatosis, steatohepatitis with or without fibrosis, fibrosis and ultimately cirrhosis, the limited data currently available in the literature can be summarized as follows. To our knowledge, one single study has analyzed OSM serum levels and the liver expression of OSM in MASLD/MASH patients stratified according to progression of the disease [67]. OSM was reported to be almost undetectable in patients with either a diagnosis of simple steatosis or of steatohepatitis. However, OSM serum levels were significantly increased in cirrhotic patients and were dramatically raised in patients with MASH-related HCC (see below), suggesting that the relevance of OSM might be related to the progression of the disease rather than the development of fatty liver and/or the early stages of MASH [67].

Along these lines, the first report examining OSM in MASLD/MASH was published almost fifteen years ago in order to investigate the contribution of this cytokine released by KCs to hepatic insulin resistance and steatosis. This experimental study, conducted both in vitro and using two different dietary protocols to induce MASLD/MASH, proposed that prostaglandin E2 (PGE2) is produced by activated KC attenuated insulin-dependent glucose utilization by interrupting the intracellular signaling cascade downstream of the insulin receptor in hepatocytes as well as by affecting insulin resistance by modulating cytokine production in non-parenchymal cells. The authors of this study showed that OSM produced by KCs exposed to PGE2 attenuated insulin-dependent Akt activation and glucokinase induction in hepatocytes through the induction of SOCS3. In addition, OSM inhibited the expression of key enzymes involved in hepatic lipid metabolism, suggesting that OSM may contribute to both the rise in IR and the development of steatosis and then possibly MASH [73]. A few years later, another study analyzed the role of OSM signaling in obesity and related metabolic disorders. The authors used mice genetically manipulated in order to carry global deletion of the OSMRβ gene (OSMRβ^−/−^ mice) and fed these mice on a high-fat diet (HFD). This study showed that OSMRβ^−/−^ mice, when compared to WT littermates, exhibited a severe increase in body weight and food intake as well as an increase in peripheral adipose tissue inflammation, insulin resistance and liver steatosis [122]. This finding was unexpected since several studies on chronic inflammatory diseases had shown that OSM inhibition was effective in preventing or attenuating disease progression [59,63,64]. In particular, OSMRβ^−/−^ mice exhibited increased expression of liver genes related to de novo lipogenesis. A major limitation of this study was that authors used mice carrying a global deletion of OSMRβ gene, resulting in the loss of its expression from all involved cells, including cells of innate (mainly monocytes/macrophages, dendritic cells and neutrophils) and adaptive (T lymphocytes) immunity as well as hematopoietic cells, mesenchymal cells as well as hepatocytes [61,62,63,64,66,67].

A more appropriate approach was reported in a study that, in addition to mice carrying global deletion of the receptor, also examined mice lacking hepatocellular OSMRβ fed on an HFD. In these mice, the dietary protocol exacerbated IR, hepatic steatosis and inflammation, effects that were markedly attenuated by the hepatocyte-specific overexpression of OSMRβ [123].

Overall, the results of these two studies should be interpreted with caution, bearing in mind that the HFD protocol is useful for reproducing steatosis but, in our experience, induces only modest inflammation and very little or negligible fibrosis. The murine HFD protocol can then, therefore, reliably reproduce just the early stage of the disease. Moreover, it should be noted these two studies did not analyze either serum or tissue levels of OSM in wild-type and genetically manipulated mice [122,123]. This is relevant since dietary protocols leading to significant inflammation and fibrosis, like high-fat/high-fructose diets or choline-deficient L-amino acid-defined (CDAA) diets, significantly up-regulated the hepatic expression of both OSM and OSMRβ at a relatively late stage, in parallel with increased collagen deposition and recruitment/activation of MFs derived from HSCs [116]. This scenario is more consistent with that described above in MASLD patients stratified by disease progression [67].

More recently, OSM has been reported to be a potential mediator of the protective effects exerted by macrophage-derived osteopontin (OPN) against the development of MASH [124]. OPN (the product of secreted phosphoprotein 1 or *SPP1* gene) is a mediator whose expression correlates with TG accumulation in MASLD patients [125] and proposed as a putative profibrogenic mediator (reviewed in ref. [126]). In this study, macrophage OPN/*SPP1* expression increased during MASH progression in humans and in mice fed on a very potent dietary protocol (high-fat/high-fructose/high-cholesterol diet), although its expression was also up-regulated in murine hepatocytes. This elegant and complex study, which used properly designed genetically modified mice, shows that a specific cluster of macrophages (i.e., of five identified), referred to as *SPP1*^high^, is formed by cells having maximal expression of OPN, which are also expressing TREM2 and CD9 antigens. This subset of macrophages did not exhibit pro-inflammatory or profibrogenic profiles but instead displayed a metabolic one. According to the study, these macrophages were responsible for MASH protection, which, in turn, was mediated by up-regulation of OSM. OSM acted on hepatocytes through STAT3 signaling, resulting in increased arginase expression. There are some comments that should be taken in mind: (i) the conclusions were mainly based on the use of genetically modified mice in which the *SPP1* gene was either overexpressed (i.e., using *SPP1* knockin mice) or deleted in myeloid cells; (ii) as suggested by the authors in their discussion, the *SPP1*^high^ TREM2 and CD9-positive macrophages may behave differently during different stages of the disease; (iii) the findings of this group [124] may be consistent with the current view that TREM2 CD9-positive cells (previously defined as LAM or scar-associated macrophages) should be interpreted as cells that, in the early stages of MASH, may act as protective cells (i.e., by aggregating and surrounding lipid-laden hepatocytes to form hepatic crown-like structures, displaying efferocytic capacity and then containing local injury and inflammation). However, under conditions of prolonged lipotoxic injury, these cells acquire a more pro-inflammatory and profibrogenic phenotype (i.e., following shedding of TREM2 and losing efferocytic capacity) [41].

### 5.3. OSM and HCC: Experimental and Clinical Data Not Directly Referring to MASLD/MASH

As mentioned in the previous section, OSM is currently believed to be a pro-carcinogenic cytokine, as observed in several human solid tumors (see Section 4.2), a view that progressively replaced the initial idea of OSM as a cytokine capable of inhibiting proliferation, which was essentially based on in vitro studies in different cancer cell lines (reviewed in refs. [61,62,63,64]). The available literature addressing OSM and HCC is relatively limited, and only recently have OSM and its related signaling axis been investigated in human patients and in mechanistic experimental models.

Interestingly, for several years, studies have investigated OSM-related effects, some of which are potentially related to a role in liver cancer, primarily by performing in vitro studies employing the widely used HepG2 human cell line. This cell line is an immortalized line of human liver cancer cells originally isolated from a liver biopsy of a 15-year-old boy with a well-differentiated hepatoblastoma, or other hepatoma cells. To our knowledge, the first study published on OSM and HepG2 cells dates back to 1991, when a biochemical article proposed that OSM was able to strongly increase the uptake of low-density lipoproteins (LDLs) through a tyrosine kinase-related mechanism, resulting in increased transcription of the LDL receptor and its increased presence on the plasma membrane [127]. These findings, which were confirmed by subsequent studies [128,129], were initially overlooked, although they conveyed a relevant message: a cytokine secreted by macrophages had effects on hepatocyte lipoprotein and cholesterol metabolism, indicating a possible connection between the immune system and cholesterol homeostasis while also demonstrating that HepG2 (i.e., liver cancer cells) expressed the receptors for OSM. The relevance of this finding is now clear since, as mentioned before and briefly discussed again below, changes in fatty acid metabolism and cholesterol pathways are particularly relevant in the pathogenesis of MASLD/MASH and MASH-related HCCs and, more generally, for liver carcinogenesis [6,10,11,12,13]. Other in vitro studies using HepG2 cells revealed a role for OSM in the regulation of the synthesis of hepatic acute-phase proteins related to coagulation, such as, for example, fibrinogen [130] and secreted phospholipase A2 [131]. They also identified a role for OSM in the up-regulation of conventional as well as soluble IL6 receptor. The latter protein lacks the transmembrane domain and has been suggested to be a potent immunomodulator of IL6 biologic activity, but once again, this finding was somewhat neglected for its putative relation to liver cancer progression [132].

In our view, the most relevant in vitro study indicated that, unlike normal hepatocytes, which express both OSM-related receptors, human hepatoma cells were characterized by a reduced expression of LIFR and reduced responsiveness to LIF. This was ascribed to epigenetic changes in hepatoma cells, resulting from the methylation-dependent inactivation of the LIFR gene. Reduced responsiveness to LIF by hepatoma cells was remarkably similar to that observed for other cancer cell lines like colon HCT8 cells, breast MCF7 cells and lung carcinoma cells [133]. If we limit the observation to hepatoma cells, the message was that liver cancer cells do not respond or minimally respond to LIF but maintain the capacity to respond to OSM through OSMRβ, suggesting that OSM can act directly on liver cancer cells.

Data more directly related to HCC were published later in an attempt to clarify the role of OSM in mediating acute-phase protein (APP) gene activation [134]. By employing mice KO for amphiregulin (AR), a ligand for epidermal growth factor receptor (EGFR) and related wild-type mice, the authors of this study found that APP genes were overexpressed in the livers of AR KO mice during inflammation and hepatocyte proliferation. Moreover, for AR-null hepatocytes in culture and human HCC cells after AR knockdown, APP gene expression is enhanced, suggesting that AR counteracts OSM-triggered STAT3 signaling in hepatocytes and attenuates APP gene transcription, supporting the relevance of epithelial growth factor receptor (EGFR)-mediated signaling in the modulation of cytokine-activated pathways. This is potentially relevant because AR, which is not expressed in the normal liver, is up-regulated during chronic liver injury and, particularly, in human HCC tissues and cell lines, where it acts as a mitogenic and antiapoptotic growth factor for HCC cells [135].

A particularly interesting study investigated OSM in relation to liver cancer stem cells (CSCs) [136] based on the evidence that IL6-related cytokines can induce the differentiation of hepatoblasts into hepatocytes [137]. In this study, OSM receptor expression was found in the majority of epithelial cell adhesion molecule (EpCAM)-positive HCC cells with stem/progenitor cell features. Interestingly, OSM induced differentiation of these cells into hepatocytes through STAT3 signaling. These EpCAM-positive cells treated with OSM showed increased cell proliferation and an expansion of the EpCAM-negative non-CSC population.

It was not until 2012 that OSM levels were reported to be 6–7-fold higher in sera of cirrhotic patients (carrying or not HCC) than in those of healthy controls. In cirrhotic patients, serum levels of OSM were significantly associated with GP73 levels; GP73 is a Golgi phosphoprotein protein considered at the time a putative biomarker for HCC [111]. To our knowledge, this study was indeed the first to report a very significant increase in serum OSM in cirrhotic patients (carrying or not HCC). Unfortunately, in this study, no information was provided regarding the specific etiology of cirrhosis [111].

In another study using different liver cancer cell lines (HepG2, Hep3B and Huh7 cells), OSM and IL6 were found to inhibit IL27-induced IFNγ-like, STAT1-dependent transcriptional response through a SOCS3-mediated mechanism [138]. The relevance of this study relies in the fact that IL27 is up-regulated in patients with HCC compared to healthy controls or patients with hepatitis and/or liver cirrhosis [139,140]. It should be mentioned that HCC is an immunosensitive tumor and that TH1 cytokine detection in patients is usually associated with a good prognosis, with type I interferons, IFNγ and IL12 being crucial for immune-mediated rejection of HCC [141]. Accordingly, IL27 has been proposed as a putative antitumor molecule with an effect mediated by increased antigen presentation but just combining the treatment with blocking antibodies against PD-L1 or/and IL6-type cytokines [142]. The overall message is that IL6-related cytokines, such as OSM, can affect the efficacy of immune responses towards liver cancer cells, a concept subsequently supported by further studies [141,142].

Finally, it is important to mention an experimental study using the N-diethylnitrosamine (DEN)-induced rat model of HCC, which revealed that the overexpression of OSM significantly increased the number of tumor nodules and shortened rat survival [143]. Moreover, OSM was reported to promote the in vivo activation of hepatic progenitor cells, to induce TNFα secretion and increase the accumulation of CD68-positive macrophages. Accordingly, the depletion of either TNFα or of macrophages is inhibited by the pro-carcinogenic effects of OSM [143].

### 5.4. OSM and OSM/OSMRβ in MASH-Related HCC

Interest in the specific role of OSM and the OSM/OSMRβ axis in MASH-related HCC is relatively recent. To our knowledge, the first clinical and experimental study to examine OSM in HCC raised in MASH patients was published in 2020 [144]. In this study, the authors analyzed two cohorts of cirrhotic and non-cirrhotic patients carrying HCC having HBV or MASLD/MASH etiology (33 HBV vs. 20 MASLD in cohort 1, 20 HBV vs. 9 MASLD in cohort 2) that were used for different tissue and experimental analyses. This study introduced a novel concept, with data that focused on the role of OSM released by neutrophils infiltrating the tumor mass [144]. This is particularly relevant because neutrophils, although less characterized than tumor-associated macrophages (TAMs) and tumor-infiltrating lymphocytes (TILs), are a significant cellular component in the HCC mass and may contribute to progression [145,146]. In summary, the study identified a relationship between the metabolic switch of macrophages toward aerobic glycolysis and their release of chemokines CXCL2 and CXCL8, which are responsible for the recruitment of neutrophils in the HCC. In addition, activated macrophages release TNFα, which, in turn, stimulate OSM production by neutrophils. This production correlates with HCC metastasis, suggesting, for the first time, OSM as a pro-metastatic mediator in HCC progression [144].

The first clinical and experimental study entirely devoted to investigating the role of OSM in the development and progression of MASH-related HCC was published in 2022 [67]. This study analyzed selected cohorts of MASLD/MASH-related HCC patients, liver cancer cell lines exposed to human recombinant OSM or stably transfected to overexpress human OSM, murine HCC xenografts, and the DEN-CDAA murine MASH-related protocol to induce experimental liver carcinogenesis. Clinically, OSM was selectively overexpressed in HCC cells from MASH patients. Moreover, the analysis of serum levels of OSM in patients, stratified according to the different stages of the disease, revealed that OSM serum levels, barely detectable in patients with steatosis or early steatohepatitis, started to increase in cirrhosis to be then strongly and significantly increased in patients carrying HCC. OSM overexpression is a selective feature of MASH-related HCC: comparing circulating OSM levels in MASH-related HCC patients with those in 51 patients with HCC developed on a different background (HBV, HCV and alcoholic liver disease), serum OSM levels were significantly higher in MASH patients than in those with other etiologies [67]. When MASH-related patients carrying HCC were divided according to the Barcelona Clinic Liver Cancer (BCLC) staging system [147], patients with intermediate or advanced HCC stages (classes B/C/D, respectively) had OSM serum levels significantly higher than those at early stages of HCC with preserved liver function (classes 0/A). Higher serum levels of OSM also correlated with poorer survival. A series of in vitro experiments using liver cancer cell lines indicated OSM overexpression or the exposure to hrOSM resulted in increased angiogenesis as well as in the induction of EMT and invasiveness, indicating that OSM up-regulation in liver cancer cells can contribute to HCC progression. This concept was reinforced by xenograft experiments in which the inoculation of cells overexpressing OSM was associated with a slower tumor growth but a markedly increased and diffuse vascularization of the tumor graft, resulting in an increased rate of lung metastasis. OSM overexpression and its positive correlation with the angiogenic switch were confirmed in the DEN-CDAA model of liver carcinogenesis and the observation that in human MASH-related HCC with vascular invasion, OSM was overexpressed in liver cancer cells invading hepatic vessels. This study also showed that liver cancer cells can both respond to and produce OSM, overall suggesting that the OSM/OSMRβ axis might support HCC progression in MASH patients [67].

In a subsequent study, the role of OSM and the OSM/OSMRβ axis in driving MASH-related HCC progression was investigated using mice carrying a hepatocyte-specific deletion of OSMRβ (hOSMRβ^−/−^ mice), together with in vitro experiments using HCC and immune cell lines and analyses of cohorts of MASH patients, either with or without HCC [83]. hOSMRβ^−/−^ mice were generated by crossing Osmrβ^fl/fl mice on a C57BL/6 background with mice carrying Cre recombinase under the control of the albumin promoter (Alb-Cre), on the same genetic background. The hepatocyte specificity of OSMRβ deletion was confirmed by comparing OSMRβ transcript levels in isolated hepatocytes and hepatic myeloid cells, as well as by assessing OSMRβ transcript and protein levels in the resulting tumors.

MASH-related hepatocarcinogenesis was induced in male hOSMRβ^−/−^ and wild-type C57BL/6 mice using the DEN-CDAA protocol. Mice received a single intraperitoneal injection of diethylnitrosamine (DEN; 25 mg/kg body weight) at two weeks of age and, starting at six weeks of age, were fed a choline-deficient, L-amino acid-defined (CDAA) diet for 26 weeks. This protocol combines DEN-mediated tumor initiation with CDAA-induced steatohepatitis and fibrosis, thereby allowing HCC to develop in a MASH-like hepatic microenvironment. Although the model does not reproduce the obesity and systemic insulin resistance typically associated with human MASH, it recapitulates major hepatic features of disease progression, including steatohepatitis, fibrosis and multifocal hepatocarcinogenesis. Two neoplastic nodules from each animal were collected for molecular and histological analyses [86].

Hepatocyte-specific OSMRβ deletion did not affect OSM production by HCCs but significantly reduced the size and weight of hepatic tumors compared with those in wild-type animals subjected to the same protocol, without affecting the overall number of macroscopically detectable nodules. This distinction suggests that hepatocellular OSMRβ signaling primarily promotes the growth and progression of established tumors rather than DEN-dependent tumor initiation. The reduction in tumor burden was associated with decreased levels of proliferation and angiogenesis markers, supporting a causal contribution of autocrine/paracrine OSM/OSMRβ signaling to the growth and vascularization of MASH-related HCC [86]. The use of genetically manipulated mice also revealed that the hepatocyte-specific deletion of the receptor did not affect OSM production by HCC but significantly reduced the size and weight of hepatic tumors compared with those detected in wild-type animals. Moreover, confirming the finding of a previous study [67], the reduction in size and weight of tumors in hOSMRβ^−/−^ mice was associated with a decrease in markers of proliferation (Ki67 and proliferating cell nuclear antigen or PCNA) as well as with a decrease in markers of angiogenesis (VEGFA, VEGF receptor 2 and CD105), indicating that autocrine/paracrine OSM/OSMRβ signaling contributes to MASH-related HCC growth and angiogenesis.

The same study subsequently showed an association between OSM expression and the tumor immune microenvironment (TIME) [86]. The hypothesis that OSM may contribute to maintaining the TIME in HCC was first introduced by a clinical study investigating a large cohort (n = 397) of HCC patients of mixed etiologies that underwent surgical resection [147]. Data reported in this study indicated that the majority of patients (n = 248) had a viral etiology (HBV or HCV), whereas for the remaining patients (n = 149), unfortunately, no specific indication of the etiology was reported. Nevertheless, the authors focused their attention on vessels encapsulating tumor cluster (VETC)-positive HCC (n = 62) and VETC negative by analyzing OSM-positive cells and inflammatory/immune cells, including CD4, CD8, CD163 and forkhead box P3 (FOXP3)-positive cells by immunohistochemistry. Data provided indicate that tumor-infiltrating OSM-positive cells were significantly low in VETC-positive HCC. Of interest, in VETC-positive HCC, characterized by a lower grade of hepatocyte differentiation, the number of OSM-positive cells was not associated with vascular invasion, whereas in VETC-negative HCC, an increase in the number of OSM-positive cells was associated with vascular invasion [147]. In a subsequent study, the same group of authors, using the same HCC liver specimens used in the previous article, reported a direct correlation between OSM, tumor CD147 expression and TILs in HCC. Authors proposed this as a mechanism by which CD147 may be able to facilitate HCC immune evasion [148], given that CD147 overexpression has been proposed as a predictor of high malignant potential and advanced clinical stage [149,150]. In this second study, authors investigated the expression of CD147 in liver tumor cells and quantified OSM-positive cells and TILs by using a microarray and immunohistochemistry approach [148]. Interestingly, most of the HCCs investigated had high CD147 expression (n = 332), and in these tumors, the density of OSM-positive cells was associated with CD8, CD4, FOXP3 and CD20-positive cells, whereas, in HCC showing low CD147 expression, OSM positivity was limited to FOXP3-positive cells.

**Figure 3:**
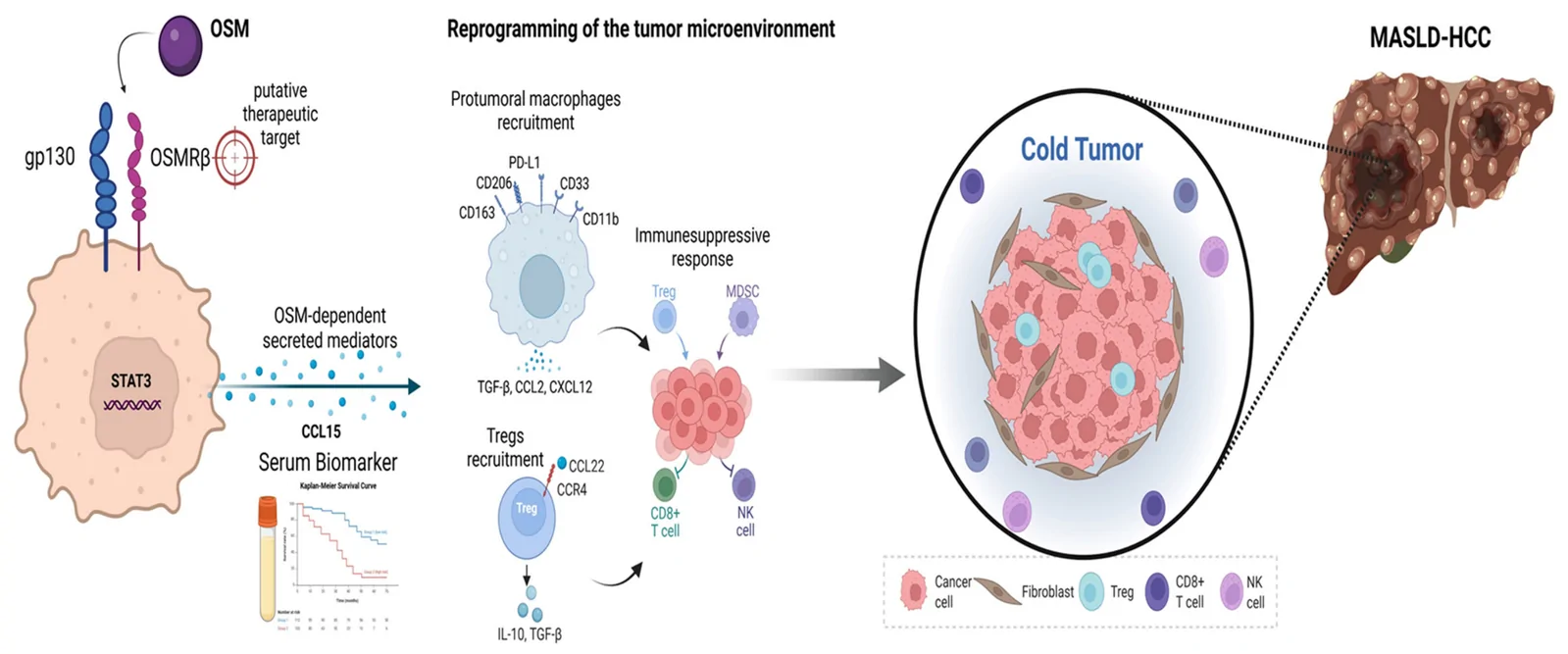
Oncostatin M (OSM)/OSMRβ signaling drives immunosuppressive reprogramming of the tumor microenvironment in MASLD-related HCC. OSM binds the heterodimeric receptor complex composed of glycoprotein 130 (gp130) and Oncostatin M receptor β (OSMRβ)—a putative therapeutic target—activating intracellular STAT3 signaling and the release of OSM-dependent secreted mediators, including CCL15, a candidate serum biomarker associated with patient survival. These mediators promote recruitment of pro-tumoral macrophages expressing CD206, CD163, CD33, CD11b and PD-L1, which release TGFβ, CCL2 and CXCL12, together with recruitment of regulatory T cells (Tregs) via the CCL22/CCR4 axis, leading to local release of IL10 and TGFβ. The resulting immunosuppressive response favors the establishment of an immune-excluded, poorly infiltrated “cold tumor” phenotype within the MASLD-HCC nodule. Created in BioRender. Cannito, S. (2026) https://BioRender.com/3ta35ma.

The concept of OSM as a mediator potentially involved in reshaping the TIME was confirmed by an analysis of data from the TCGA-LIHC cohort, which includes HCC patients of different etiologies [86] (Figure 3). This analysis identified a significant positive correlation between OSM expression and one of several genes that the literature associates with TIME, such as TAMs and regulatory immune cells markers (CD206, CD163, PD-L1, TGFβ1, CCL22, CCL2, FOXP3, IL2RA, CXCL12, CD33, CD11b, and cyclooxygenase-2). An almost overlapping pattern was detected in HCCs obtained in the liver of mice submitted to the DEN-CDAA protocol of MASH-related carcinogenesis, with OSM expression correlating with either CCL2, TGFβ1, and CXCL12 (i.e., involved in TAMs recruitment) or PD-L1, prostaglandin E synthase 2 (PTGSE2), and CD206 (TAM-associated markers) as well as FOXP3, IL2 receptor antagonist (IL2-RA), CCL22 and CC chemokine receptor (CCR) 4 (markers of regulatory T cells or Treg). Accordingly, all these cytokines, chemokines and markers were significantly down-regulated in HCC from hOSMRβ^−/−^ mice and this pattern was associated with reduced OSM-related signaling (i.e., involving STAT3 phosphorylation) [86]. Finally, a single-cell RNA-seq analysis of human HCCs identified liver cancer cells as the source of the chemokine CCL15, which has been reported to be a putative HCC biomarker associated with immunosuppression in HCCs. Serum levels of CCL15 were strongly up-regulated in both humans and mice carrying MASH-related HCCs, and Kaplan–Meier analysis showed that high CCL15 serum levels were correlated with significantly worse survival of human patients [86]. Moreover, CCL15 serum levels were significantly higher in MASH patients carrying HCCs than in patients carrying HCCs of different etiology. These data are consistent with those previously reported by another research group that suggested CCL15 as a putative HCC biomarker whose expression is significantly correlated with malignant HCC behavior and poor patient survival [151]. Related in vitro studies confirmed that by blocking autocrine OSM signaling in HepG2 or Huh7 cells, overexpressing OSM reduced STAT3 phosphorylation, CCL15 production, and prevented TIME marker expression by co-cultured macrophage-derived THP-1 cells, indicating a direct relationship between OSM and the up-regulation of CCL15.

## 6. Conclusions

Although OSM is also involved in CLD progression and HCC development of different etiologies, OSM and the OSM/OSMRβ axis have an emerging and apparently more relevant role in MASLD/MASH. In concluding this review, we propose that OSM, and the OSM/OSMRβ axis and strictly related mediators, may work differently in relation to the stage and progression of the disease. According to the data and studies mentioned in this review, we propose the following general interpretation in relation to disease progression.

Early stages of the disease. Based on some mechanistic studies indicating that OSMRβ deletion may exacerbate early metabolic dysfunction (i.e., steatosis), OSM in the early metabolic stage is likely to act as a context-dependent mediator exerting regulatory, possibly protective action.

Disease progression. During disease progression and then in relation to the metabolically related prolonged lipotoxic injury, OSM becomes a pro-inflammatory and profibrogenic mediator capable of contributing significantly to disease progression. Based on the evidence discussed above (see Section 5.2), it seems reasonable to speculate that this dual role of OSM (potentially protective in the early stages but unequivocally pro-inflammatory and pro-fibrogenic in advanced stages) may be closely related to that described for TREM2 CD9-positive macrophages that, during disease progression, turn form protective cells at early stages into pro-inflammatory and profibrogenic cells. This specific aspect should be further and carefully investigated in future studies.

MASH-related HCC. Increased OSM expression is emerging as a particularly relevant feature of HCC with a metabolic etiology and correlates with clinical parameters and disease outcomes. Data from patients and murine studies as well as from in vitro studies suggest that OSM and its signaling have a pro-angiogenic role and can efficiently sustain the proliferation, EMT and increased invasiveness of liver cancer cells. Moreover, OSM is now suggested to have a role in reshaping TIME and then favoring immune evasion of cancer cells in HCC. This promising and clinically relevant hypothesis should be tested in the future to validate the synergy between OSM inhibition and ICI efficacy. Overall, OSM can be proposed as a candidate biomarker and a putative therapeutic target for MASH-related HCC. The most relevant pre-clinical and clinical data supporting the involvement of OSM and the OSM/OSMRβ axis in CLD, MASLD/MASH and HCC cited in this review are summarized in Table 1.

## Figures and Tables

**Table 1:** Experimental and clinical evidence supporting the involvement of OSM/OSMRβ signaling in Chronic Liver Disease, MASLD/MASH and HCC. The table summarizes the main clinical and experimental studies discussed in Section 5.1, Section 5.2, Section 5.3 and Section 5.4, organized according to disease context. For each study, the model or system employed, the assessment or experimental manipulation of the OSM/OSMRβ axis, the main cellular sources and targets, the principal findings, and the translational relevance and limitations are reported. Abbreviations: CDAA, choline-deficient L-amino acid-defined diet; CLD, Chronic Liver Disease; DEN, diethylnitrosamine; HCC, hepatocellular carcinoma; HFD, high-fat diet; HSC, hepatic stellate cells; LIFRβ, leukemia inhibitory factor receptor β; MASLD, metabolic dysfunction-associated steatotic liver disease; MASH, metabolic dysfunction-associated steatohepatitis; OSM, oncostatin M; OSMRβ, oncostatin M receptor β; TAA, thioacetamide; TAMs, tumor-associated macrophages; TIME, tumor immune microenvironment; Tregs, regulatory T cells.

Disease Context	Model/System	OSM/OSMRβ Assessment or Manipulation; Source/Target Cells	Main Findings	Human Disease Features Reproduced and Translational Limitations	Ref.
**Chronic Liver Disease**					
Human cirrhosis	Human cirrhotic liver specimens versus unused donor livers	Immunohistochemical analysis of OSM, OSMRβ and LIFRβ; CD68^+^ macrophages/Kupffer cells identified as the main OSM-expressing population	OSM expression was higher in cirrhotic than control livers. OSMRβ expression was low in hepatocytes and apparently unchanged, whereas LIFRβ was increased in cholangiocytes of reactive ductules	Direct evidence from human liver tissue; however, the cohort was small, and the etiology of cirrhosis was not reported	[102]
Liver fibrogenesis	Human activated HSC-derived liver myofibroblasts	Exogenous OSM stimulation; activated HSC/myofibroblasts as target cells	OSM increased collagen type I and TIMP1 expression, providing early evidence of a direct profibrogenic action	Human cell-based system relevant to fibrogenesis, but unable to reproduce multicellular interactions and the chronic liver microenvironment	[103,111]
Chronic toxic liver injury and regeneration	OSMRβ-knockout mice exposed to CCl_4_; partial hepatectomy	Genetic deletion of OSMRβ; hepatocytes and cells involved in tissue remodelling as targets	OSMRβ deficiency impaired hepatocyte proliferation and tissue remodelling and reduced TIMP1 and TIMP2 expression	Reproduces toxic injury and regenerative/remodelling responses, but CCl_4_ does not reproduce the metabolic and immunological complexity of human MASLD/MASH	[104]
Liver progenitor-cell response and regeneration	Rat 2-acetylaminofluorene/partial-hepatectomy model; oval-cell cultures	Endogenous OSM detected in Kupffer and oval cells; OSM receptor mainly expressed by oval cells	OSM inhibited oval-cell proliferation and promoted their differentiation toward hepatocytes	Useful for studying progenitor-cell activation and regeneration; limited relevance to the mechanisms driving chronic human CLD and MASH	[105]
Toxic liver injury	Rat dimethylnitrosamine-induced liver-injury model; OSM gene delivery	Increased OSM expression; hepatocytes as principal target cells	OSM promoted hepatocyte proliferation and survival and attenuated liver injury	Models toxic injury and hepatocyte repair, but not metabolic liver disease; gene delivery may produce non-physiological OSM exposure	[106]
Angiogenic response	Human HepG2 and PH5CH8 cells	Recombinant OSM stimulation; hepatocyte-derived cells as targets	OSM induced HIF-1α through JAK/STAT3 and ERK1/2 signaling, increasing VEGF and PAI-1 expression	Identifies a mechanistic connection between OSM, hypoxia signaling and angiogenesis; limited by the use of immortalized cell lines and absence of the tissue microenvironment	[107]
Cirrhosis/HCC biomarker-related response	Human HepG2 cells; sera from patients with liver disease	OSM or IL-6 stimulation; measurement of circulating OSM and GP73	OSM strongly induced GP73 in HepG2 cells. Serum OSM was increased in patients with cirrhosis, with or without HCC, and was associated with GP73 levels	Includes clinical evidence, but disease etiology was not reported and the study could not establish whether OSM elevation was specific to a particular CLD or HCC background	[108]
Chronic liver fibrosis	OSM-knockout mice exposed to TAA for 12 weeks; HSC–macrophage co-cultures; hydrodynamic OSM overexpression	Genetic OSM deletion, recombinant OSM stimulation and sustained OSM overexpression; recruited macrophages as source and HSC as targets	OSM deletion reduced fibrosis, collagen 1a1 and TIMP1 without reducing hepatocyte injury. OSM promoted macrophage–HSC cooperation and increased PDGF, TGF-β and TIMP1; sustained OSM expression was sufficient to induce fibrosis	Provides strong mechanistic evidence for OSM-driven fibrogenesis; however, TAA causes toxic injury and does not reproduce the metabolic basis of human MASLD/MASH, while hydrodynamic overexpression may generate supraphysiological OSM levels	[113]
Fibrogenesis in MASLD/MASH	Human LX2 myofibroblast-like cells; human MASLD specimens; HFD/high-fructose and CDAA mouse models	Human recombinant OSM stimulation; HSC-derived myofibroblasts as target cells; assessment of hepatic OSM/OSMRβ expression	OSM induced LX2 chemotaxis through ROS, STAT1/STAT3, HIF-1α and VEGF and increased CCL2, IL-6 and TNF-α. Hepatic OSM and OSMRβ increased with inflammation, fibrosis and myofibroblast activation	Connects human-cell mechanisms with human disease and dietary models. CDAA efficiently induces inflammation and fibrosis but causes weight loss and lacks the obesity/insulin-resistance phenotype typical of human MASH; HFD/high-fructose models reproduce metabolic dysfunction but generally develop less severe fibrosis	[114]
Advanced fibrosis of mixed etiology	Human liver specimens spanning fibrosis stages F1–F4	Hepatic gene-expression analysis; OSM included in a multigene immune signature	An OSM-containing immune signature discriminated mild from advanced fibrosis independently of several clinicopathological features; expression of the signature declined at advanced stages	Direct clinical relevance across fibrosis stages, but the mixed etiologies limit conclusions regarding disease-specific OSM regulation	[115]
Cirrhosis and liver failure	Rat chronic CCl_4_ model	Analysis of cytokine expression and cellular sources/targets; Kupffer cells, macrophages and endothelial cells as OSM sources; hepatocytes, HSC and endothelial cells as targets	OSM and IL-1β were among the most strongly up-regulated cytokines, supporting broad paracrine signaling among inflammatory, parenchymal, mesenchymal and endothelial cells	Reproduces fibrosis, cirrhosis and functional deterioration; however, CCl_4_-dependent toxic injury differs from human metabolic liver disease and may overemphasize centrilobular necroinflammation	[116]
Early alcohol-associated liver disease	Gao-binge diet in YAP-deficient, hepatocyte-specific YAP-deficient and YAP-overexpressing mice	Modulation of hepatocellular YAP; hepatocytes as targets of OSM/STAT3 signaling	Hepatocellular YAP negatively regulated OSM/STAT3-dependent CD36 expression. YAP deficiency worsened steatosis and inflammation, whereas YAP overexpression was protective	Relevant to early alcohol-associated steatosis and inflammation, but does not model advanced fibrosis or MASLD/MASH and addresses OSM indirectly through YAP modulation	[117]
**MASLD/MASH**					
MASLD/MASH progression	Human patients with steatosis, steatohepatitis, cirrhosis or MASH-related HCC	Measurement of circulating OSM and analysis of hepatic OSM expression	OSM was almost undetectable in steatosis and early steatohepatitis, increased in cirrhosis and markedly elevated in MASH-related HCC, suggesting an association with disease progression rather than initial steatosis	Direct human evidence across disease stages; cohort size and cross-sectional design do not establish a causal role for OSM	[64]
Hepatic insulin resistance and steatosis	In vitro Kupffer cell/hepatocyte systems and two dietary mouse models of steatosis/MASH	PGE2-induced OSM production by Kupffer cells; hepatocytes as OSM targets	Kupffer cell-derived OSM induced SOCS3, attenuated insulin-dependent Akt activation and glucokinase induction, and inhibited enzymes involved in hepatic lipid metabolism	Reproduces Kupffer cell–hepatocyte crosstalk and metabolic dysfunction; dietary models mainly reflect early disease and do not fully reproduce progressive fibrotic human MASH	[120]
Obesity-associated steatosis and insulin resistance	Global OSMRβ-knockout mice fed an HFD	Systemic genetic deletion of OSMRβ	OSMRβ deficiency increased food intake, body weight, adipose-tissue inflammation, insulin resistance, hepatic steatosis and hepatic expression of genes involved in de novo lipogenesis	Reproduces obesity, insulin resistance and steatosis; global receptor deletion prevents attribution of the phenotype to hepatocytes and affects immune, hematopoietic and stromal compartments. HFD induces limited hepatic inflammation and negligible fibrosis	[121]
Obesity-associated steatosis and insulin resistance	Global and hepatocyte-specific OSMRβ-deficient mice fed an HFD; hepatocyte-specific OSMRβ overexpression	Cell-specific deletion or overexpression of OSMRβ in hepatocytes	Hepatocyte-specific OSMRβ deficiency exacerbated insulin resistance, steatosis and inflammation, whereas hepatocellular OSMRβ overexpression attenuated these alterations	Supports a hepatocyte-specific protective metabolic function of OSMRβ. However, HFD mainly reproduces early MASLD and does not model advanced fibrotic MASH; circulating and hepatic OSM levels were not assessed	[122]
Progressive experimental MASH and fibrosis	HFD/high-fructose and CDAA mouse models; human MASLD samples and LX2 cells	Assessment of hepatic OSM/OSMRβ; recombinant human OSM stimulation of HSC-derived myofibroblasts	Hepatic OSM and OSMRβ increased at later stages in parallel with inflammation, collagen deposition and HSC-derived myofibroblast recruitment. OSM directly promoted myofibroblast migration and inflammatory mediator expression	HFD/high-fructose models reproduce metabolic dysfunction but generally develop limited fibrosis. CDAA induces robust steatohepatitis and fibrosis but lacks obesity and systemic insulin resistance and can cause weight loss	[114]
Macrophage-mediated protection during MASH	Human MASH samples; high-fat/high-fructose/high-cholesterol-fed mice; SPP1-knock-in mice and mice with myeloid SPP1 deletion	OSM induction by SPP1high/TREM2^+^/CD9^+^ macrophages; hepatocytes as targets through STAT3	SPP1high macrophage-derived OSM activated hepatocyte STAT3 and arginase expression and contributed to protection against MASH	Reproduces several metabolic, inflammatory and histological aspects of MASH and uses cell-specific genetic approaches. However, the conclusions depend substantially on experimental SPP1 manipulation, and TREM2^+^/CD9^+^ macrophages may shift from protective to profibrogenic phenotypes during disease progression	[123]
**HCC not specifically associated with MASLD/MASH**					
Lipid metabolism in liver cancer cells	Human HepG2 cells	Exogenous OSM; hepatoma cells as OSM-responsive targets	OSM increased LDL-receptor transcription, membrane expression and LDL uptake through tyrosine kinase-dependent signaling	Demonstrates functional OSM responsiveness and a link with cholesterol metabolism; HepG2 cells derive from hepatoblastoma rather than conventional adult HCC and cannot reproduce the tumor microenvironment	[126,127,128]
Acute-phase and inflammatory responses	Human HepG2 or other hepatoma cells	Exogenous OSM stimulation	OSM regulated fibrinogen and secreted phospholipase A2 expression and increased conventional and soluble IL-6 receptor expression	Mechanistic evidence of OSM responsiveness in liver cancer-derived cells; indirect relationship with HCC biology and absence of stromal and immune components	[129,130,131]
Differential receptor usage in liver cancer cells	Human hepatoma and non-malignant hepatocyte-derived cells	Analysis of LIFR and OSMRβ-dependent responsiveness	Hepatoma cells showed epigenetic down-regulation of LIFR and reduced responsiveness to LIF while retaining responsiveness to OSM through OSMRβ	Supports preferential dependence of liver cancer cells on OSMRβ signaling; mainly based on cultured cell lines and not directly validated in HCC tissues	[132]
Acute-phase response in HCC	Amphiregulin-knockout mice, primary hepatocytes and human HCC cells with amphiregulin knockdown	Interaction between amphiregulin/EGFR and OSM/STAT3 signaling	Amphiregulin counteracted OSM-triggered STAT3 activation and acute-phase gene expression in hepatocytes and HCC cells	Identifies crosstalk between EGFR and OSM pathways; acute-phase gene regulation is only indirectly related to tumor progression, and germline amphiregulin deletion has systemic effects	[133]
Liver cancer stem/progenitor cells	EpCAM^+^ human HCC cells	OSM stimulation; OSMR-expressing EpCAM^+^ cancer stem/progenitor cells as targets	OSM induced STAT3-dependent hepatocytic differentiation, increased proliferation and expanded the EpCAM-negative non-CSC population	Relevant to tumor-cell plasticity and differentiation; ex vivo/in vitro conditions do not reproduce the immune or stromal tumor microenvironment	[135]
Cirrhosis and HCC	Human sera; HepG2 cells	Measurement of circulating OSM and GP73; OSM stimulation in vitro	Serum OSM was increased in cirrhotic patients with or without HCC and correlated with GP73; OSM strongly induced GP73 in HepG2 cells	Provides clinical and mechanistic evidence, but HCC etiology was not reported and the study could not establish disease specificity or causality	[108]
Suppression of antitumor cytokine signaling	HepG2, Hep3B and Huh7 cells	OSM or IL-6 stimulation during IL-27 exposure	OSM induced SOCS3 and inhibited IL-27-mediated STAT1/IFNγ-like transcriptional responses, suggesting attenuation of antitumor immune signaling	Mechanistically relevant to tumor immune escape, but based on tumor-cell monocultures without immune effector cells	[137]
Experimental hepatocarcinogenesis	DEN-induced HCC in rats	OSM overexpression; hepatic progenitor cells, macrophages and TNF-α-dependent signaling	OSM increased tumor-nodule number, activated hepatic progenitor cells, increased TNF-α and CD68^+^ macrophage accumulation and shortened survival. TNF-α or macrophage depletion attenuated its pro-carcinogenic effects	Reproduces multistep chemical hepatocarcinogenesis and macrophage–tumor crosstalk; DEN-driven mutagenesis does not reproduce metabolic MASH-HCC, and OSM overexpression may exceed physiological levels	[142]
**MASH-related HCC**					
HCC of HBV or MASLD/MASH etiology	Human cirrhotic and non-cirrhotic HCC specimens; tumor-derived monocytes/macrophages and neutrophils	Glycolytically activated macrophages released CXCL2/CXCL8 and TNF-α; TNF-α induced OSM production by tumor-infiltrating neutrophils	Neutrophil-derived OSM was associated with HCC metastasis, identifying an immune-cell circuit linking macrophage metabolism, neutrophil recruitment and tumor progression	Direct evidence from human HCC, including MASH-HCC; however, the cohort combined HBV and metabolic etiologies and did not isolate an exclusively MASH-specific mechanism	[143]
MASH-related HCC progression	Human MASLD/MASH-HCC cohorts; liver cancer cell lines treated with recombinant human OSM or engineered to overexpress OSM; murine xenografts; DEN-CDAA model	Measurement and experimental overexpression of OSM; HCC cells as both OSM sources and OSMRβ-expressing targets	OSM was selectively elevated in MASH-HCC and increased with disease stage. OSM promoted angiogenesis, EMT and invasiveness. OSM-overexpressing xenografts showed increased vascularization and lung metastasis, while OSM expression correlated with the angiogenic switch in DEN-CDAA tumors	Integrates human, cellular and in vivo evidence. Xenografts lack an intact immune system, whereas DEN-CDAA reproduces steatohepatitis, fibrosis and HCC but combines a chemical initiator with a choline-deficient diet and does not reproduce obesity or systemic insulin resistance	[64]
OSMRβ-dependent MASH-HCC growth and immune remodelling	DEN-CDAA-treated mice with hepatocyte-specific OSMRβ deletion; human MASH-HCC cohorts; TCGA-LIHC; HepG2/Huh7 and THP-1 co-cultures	Hepatocyte-specific OSMRβ deletion or blockade of autocrine OSM signaling; liver cancer cells as OSM-responsive targets and source of CCL15; macrophages/Tregs as downstream immune targets	OSMRβ deletion reduced tumor size and weight, proliferation, angiogenesis, STAT3 activation and immunosuppressive mediators. OSM/OSMRβ signaling induced CCL15 and promoted TAM- and Treg-associated signatures, including CD206, CD163, PD-L1, TGF-β1, CCL2, CCL22/CCR4 and FOXP3	Provides cell-specific genetic evidence and integrates mouse, human and in vitro data. DEN-CDAA does not reproduce obesity and insulin resistance, and TCGA-LIHC includes mixed etiologies. Cross-species differences in OSM receptor usage must be considered	[83]
Immunosuppressive microenvironment in HCC	Surgically resected human HCC cohort of mixed or incompletely reported etiology; VETC-positive and VETC-negative tumors	Immunohistochemical analysis of OSM^+^ cells, CD4^+^, CD8^+^, CD163^+^ and FOXP3^+^ cells	OSM^+^ infiltrating cells were less abundant in VETC-positive HCC. In VETC-negative tumors, increased OSM positivity was associated with vascular invasion	Large human cohort and direct tumor-microenvironment assessment; predominantly viral or unspecified etiologies limit extrapolation to MASH-HCC, and the observational design cannot establish causality	[146]
OSM, CD147 and tumor-infiltrating lymphocytes	Human HCC tissue microarrays from the same clinical series	Immunohistochemical analysis of tumor CD147, OSM^+^ cells and TILs	In CD147-high tumors, OSM^+^ cell density was associated with CD8^+^, CD4^+^, FOXP3^+^ and CD20^+^ infiltrates; in CD147-low tumors, the association was mainly restricted to FOXP3^+^ cells	Supports an association between OSM, CD147 and immune-cell composition, but uses an overlapping cohort, includes mixed/unspecified etiologies and remains correlative	[147]

## Data Availability

No new data were created or analyzed in this study. Data sharing is not applicable to this article.

## Abbreviations

The following abbreviations are used in this manuscript:

ALD	Alcohol-related Liver Disease
αSMA	α-Smooth Muscle Actin
APOB	Apolipoprotein B
APP	Acute-Phase Protein
AR	Amphiregulin
BCLC	Barcelona Clinic Liver Cancer
bFGF	basic Fibroblast Growth Factor
BMI	Body Mass Index
CCL	CC-chemokine Ligand
CCL_4_	Carbon tetrachloride
CCR	CC-chemokine Receptor
CD	Cluster Designation
CDAA	choline-deficient L-amino acid–defined
C/EBP	CCAAT-enhancer-binding proteins
ChREBP	Carbohydrate Response Element Binding-Protein
CLD	Chronic Liver Disease
COL13A1	Collagen type XIII Alpha 1 chain
CPT1	Carnitine O-PalmitoylTransferase
CSCs	Cancer Stem Cells
CTGF	Connective Tissue Growth Factor
CXCL	CXC-chemokine Ligand
DAMPs	Damage Associated Molecular Patterns
DEN	N-diethyl-nitrosamine
DG	DiacylGlycerol
DGAT2	DiacylGlycerol O-AcylTransferase 2
DNL	De Novo Lipogenesis
ECM	ExtraCellular Matrix
EFCAB4B	EF-Hand Calcium Binding Domain 4B
EGFR	Epithelial Growth Factor Receptor
EMT	Epithelial-to-Mesenchymal Transition
EpCAM	Epithelial Cell Adhesion Molecule
ER	Endoplasmic Reticulum
ERK	Extracellular Regulated Kinase
EV	Extracellular Vesicles
FA	Fatty Acids
FDFT1	Farnesyl-Diphosphate FarnesylTransferase 1
FFA	Free Fatty Acids
FOXP3	Forkhead box P3
GCKR	Glucokinase Receptor
GP73	GlycoProtein 73
gp130	glycoprotein 130
HCC	Hepatocellular carcinoma
HDL	High Density Lipoproteins
HSC	Hepatic Stellate Cells
HFD	High Fat Diet
HIF	Hypoxia-Inducible Factor
IBD	Inflammatory Bowel Disease
ICI	Immune-Checkpoint Inhibitors
IFNγ	Interferon γ
IFNL4	Interferon Lambda 4
IL	Interleukin
IL1β	Interleukin 1β
IL2RA	IL2 receptor antagonist
IR	Insulin Resistance
JAK	Janus Kinases
JNK	c-Jun NH2 aminoterminal Kinase
KC	Kupffer Cells
LAMs	Lipid-Associated Macrophages
LDL	Low Density Lipoprotein
LIF	Leukemia Inhibitory Factor
LIFRβ	LIF Receptor β
LXR	Liver X Receptors
MAPK	Mitogen-Associated Protein Kinase
MASH	Metabolic dysfunction—Associated SteatoHepatitis
MASLD	Metabolic dysfunction-associated steatotic liver disease
MBOAT7	Membrane Bound O-AcylTransferase domain-containing 7
MFs	MyoFibroblasts
MMPs	Matrix MetalloProteases
MoMs	Monocyte-derived macrophages
MTTP	Microsomal Triglycerides Transfer Protein
NK	Natural Killer cells
NKT	Natural Killer T cells
OPN	Osteopontin
OSM	Oncostatin M
OSMRβ	Oncostatin M Receptor β
PAMPs	Pathogen-Associated Molecular Patterns
PCNA	Proliferating Cell Nuclear Antigen
PDGF	Platelet-Derived Growth Factor
PD-L1	Programmed Death-Ligand 1
PGE2	Prostaglandin E2
PTGSE2	Prostaglandin E synthase 2
PI3K	Phosphatidyl Inositol 3 Kinase
PNPLA3	Patatin-like PhosphoLipase domain containing 3
PPARγ	Peroxisome Proliferator-Activated Receptor γ
PRR	Pattern Recognition Receptors
ROS	Reactive Oxygen Species
SEC	Sinusoidal Endothelial Cells
SREBP1c	Sterol Regulatory Element Binding-Protein 1c
SOCS	Suppressors Of Cytokine Signaling
SOD	SuperOxide Dismutase
*SPP1*	Secreted PhosphoProtein 1 gene
STAT	Signal Transducer and Activator of Transcription
TAA	Thioacetamide
TACE	TransArterial ChemoEmbolization
TAMs	Tumor Associated Macrophages
TG	Triglycerides
TGFα	Transforming Growth Factor α
TGFβ1	Transforming Growth Factor β1
TH1	T Helper 1
TILs	Tumor Infiltrating Lymphocytes
TIME	Tumor Immunosuppressive MicroEnvironment
TIMPs	Tissue Inhibitors of MetalloProteases
TM6SF2	TransMembrane 6 SuperFamily member 2
TNFα	Tumor Necrosis Factor α
T2D	Type 2 Diabetes
Treg	regulatory T cells
TREM2	Triggering Receptor Expressed on Myeloid cells 2
TYK2	Tyrosine Kinase 2
UCP2	Uncoupling Protein 2
VETC	Vessels Encapsulating Tumor Cluster
VEGF	Vascular Endothelial Growth Factor
VLDL	Very Low Density Liporoteins
WAT	White Adipose Tissue
YAP	Yes-Associated Protein

## References

[1] Golabi P., Isakov V., Younossi Z.M. (2023). Nonalcoholic fatty liver disease: Disease burden and disease awareness. Clin. Liver Dis..

[2] Estes C., Anstee Q.M., Arias-Loste M.T., Bantel H., Bellentani S., Caballeria J., Colombo M., Craxi A., Crespo J., Day C.P. (2018). Modeling NAFLD disease burden in China, France, Germany, Italy, Japan, Spain, United Kingdom, and United States for the period 2016–2030. J. Hepatol..

[3] Byrne C.D., Armandi A., Pellegrinelli V., Vidal-Puig A., Bugianesi E. (2025). Μetabolic dysfunction-associated steatotic liver disease: A condition of heterogeneous metabolic risk factors, mechanisms and comorbidities requiring holistic treatment. Nat. Rev. Gastroenterol. Hepatol..

[4] Rinella M.E., Lazarus J.V., Ratziu V., Francque S.M., Sanyal A.J., Kanwal F., Romero D., Abdelmalek M.F., Anstee Q.M., Arab J.P. (2023). NAFLD Nomenclature consensus group. A multi-society Delphi consensus statement on new fatty liver disease nomenclature. J. Hepatol..

[5] Chalasani N., Younossi Z., Lavine J.E., Charlton M., Cusi K., Rinella M., Harrison S.A., Brunt E.M., Sanyal A.J. (2018). The diagnosis and management of nonalcoholic fatty liver disease: Practice guidance from the American Association for the Study of Liver Diseases. Hepatology.

[6] Powell E.E., Wong V.W., Rinella M. (2021). Non-alcoholic fatty liver disease. Lancet.

[7] Huang D.Q., El-Serag H.B., Loomba R. (2021). Global epidemiology of NAFLD-related HCC: Trends, predictions, risk factors and prevention. Nat. Rev. Gastroenterol. Hepatol..

[8] Singal A.G., Kanwal F., Llovet J.M. (2023). Global trends in hepatocellular carcinoma epidemiology: Implications for screening, prevention and therapy. Nat. Rev. Clin. Oncol..

[9] Mauro E., de Castro T., Zeitlhofer M., Sung M.W., Villanueva A., Mazzaferro V., Llovet J.M. (2025). Hepatocellular carcinoma: Epidemiology, diagnosis and treatment. JHEP Rep..

[10] Parola M., Pinzani M. (2024). Liver fibrosis in NAFLD/NASH: From pathophysiology towards diagnostic and therapeutic strategies. Mol. Asp. Med..

[11] Geng Y., Liu L., Sun Y., Guo L., Wu Y., Jia Z. (2025). Metabolic dysregulation in MASLD-associated HCC: Diagnostic biomarkers and therapeutic opportunities. Front. Med..

[12] Rinella M.E., Sookoian S. (2026). Therapeutic targets for metabolic dysfunction associated steatohepatitis: A personalized approach to disease management. Nat. Rev. Gastroenterol. Hepatol..

[13] Pinto E., Meneghel P., Farinati F., Russo F.P., Pellizzaro F., Gambato M. (2024). Efficacy of immunotherapy in hepatocellular carcinoma: Does liver disease etiology have a role?. Dig. Liver Dis..

[14] Li X., Jin K., Fang J., Ma X., He J., Lu J., Jia Z., Shi A. (2026). Immune crosstalk in metabolic dysfunction-associated steatotic liver disease: Interactions between innate and adaptive immunity. Front. Immunol..

[15] Loomba R., Friedman S.L., Shulman G.I. (2021). Mechanisms and disease consequences of nonalcoholic fatty liver disease. Cell.

[16] Piccinin E., Villani G., Moschetta A. (2026). Cholesterol-LXR axis in metabolic regulation of liver fibrosis and hepatocarcinogenesis. Trends Endocrinol. Metab..

[17] Wang B., Tontonoz P. (2018). Liver X receptors in lipid signalling and membrane homeostasis. Nat. Rev. Endocrinol..

[18] Bovenga F., Sabbà C., Moschetta A. (2015). Uncoupling nuclear receptor LXR and cholesterol metabolism in cancer. Cell Metab..

[19] Schultz J.R., Tu H., Luk A., Repa J.J., Medina J.C., Li L., Schwendner S., Wang S., Thoolen M., Mangelsdorf D.J. (2000). Role of LXRs in control of lipogenesis. Genes Dev..

[20] Ibrahim S.H., Hirsova P., Gores G.J. (2018). Non-alcoholic steatohepatitis pathogenesis: Sublethal hepatocyte injury as a driver of liver inflammation. Gut.

[21] Hirsova P., Gores G.J. (2015). Death receptor-mediated cell death and proinflammatory signaling in nonalcoholic steatohepatitis. Cell. Mol. Gastroenterol. Hepatol..

[22] Schuster S., Cabrera D., Arrese M., Feldstein A.E. (2018). Triggering and resolution of inflammation in NASH. Nat. Rev. Gastroenterol. Hepatol..

[23] Knorr J., Wree A., Feldstein A.E. (2022). Pyroptosis in steatohepatitis and liver diseases. J. Mol. Biol..

[24] Horn P., Tacke F. (2024). Metabolic reprogramming in liver fibrosis. Cell Metab..

[25] Marra F., Svegliati-Baroni G. (2018). Lipotoxicity and the gut-liver axis in NASH pathogenesis. J. Hepatol..

[26] Hirsova P., Ibrabim S.H., Gores G.J., Malhi H. (2016). Lipotoxic lethal and sublethal stress signaling in hepatocytes: Relevance to NASH pathogenesis. J. Lipid Res..

[27] Povero D., Eguchi A., Niesman I.R., Andronikou N., de Mollerat du Jeu X., Mulya A., Berk M., Lazic M., Thapaliya S., Parola M. (2013). Lipid-induced toxicity stimulates hepatocytes to release angiogenic microparticles that require Vanin-1 for uptake by endothelial cells. Sci. Signal..

[28] Povero D., Panera N., Eguchi A., Johnson C.D., Papouchado B.G., de Araujo Horcel L., Pinatel E.M., Alisi A., Nobili V., Feldstein A.E. (2015). Lipid-induced hepatocyte-derived extracellular vesicles regulate hepatic stellate cell via microRNAs targeting PPAR-γ. Cell. Mol. Gastroenterol. Hepatol..

[29] Cannito S., Morello E., Bocca C., Foglia B., Benetti E., Novo E., Chiazza F., Rogazzo M., Fantozzi R., Povero D. (2017). Microvesicles released from fat-laden cells promote activation of hepatocellular NLRP3 inflammasome: A pro-inflammatory link between lipotoxicity and non-alcoholic steatohepatitis. PLoS ONE.

[30] Anstee Q.M., Seth D., Day C.P. (2016). Genetic factors that affect risk of alcoholic and nonalcoholic fatty liver disease. Gastroenterology.

[31] Eslam M., George J. (2020). Genetic contributions to NAFLD: Leveraging shared genetics to uncover systems biology. Nat. Rev. Gastroenterol. Hepatol..

[32] Eslam M., Valenti L., Romeo S. (2018). Genetics and epigenetics of NAFLD and NASH: Clinical impact. J. Hepatol..

[33] Trépo E., Valenti L. (2020). Update on NAFLD genetics: From new variants to the clinic. J. Hepatol..

[34] Sookoian S., Rotman Y., Valenti L. (2024). Genetics of Metabolic Dysfunction-associated Steatotic Liver Disease: The state of the art update. Clin. Gastroenterol. Hepatol..

[35] Schwabe R.F., Tabas I., Pajvani U.B. (2020). Mechanisms of fibrosis development in nonalcoholic steatohepatitis. Gastroenterology.

[36] Peiseler M., Schwabe R., Hampe J., Kubes P., Heikenwalder M., Tacke F. (2022). Immune mechanisms linking metabolic injury to inflammation and fibrosis in fatty liver disease—Novel insights into cellular communication circuits. J. Hepatol..

[37] Wallace S.J., Tacke F., Schwabe R.F., Henderson N.C. (2022). Understanding the cellular interactome of non-alcoholic fatty liver disease. JHEP Rep..

[38] Ramachandran P., Matchett K.P., Dobie R., Wilson-Kanamori J.R., Henderson N.C. (2020). Single-cell technologies in hepatology: New insights into liver biology and disease pathogenesis. Nat. Rev. Gastroenterol. Hepatol..

[39] Saviano A., Henderson N.C., Baumert T.F. (2020). Single-cell genomics and spatial transcriptomics: Discovery of novel cell states and cellular interactions in liver physiology and disease biology. J. Hepatol..

[40] Sutti S., Albano E. (2020). Adaptive immunity: An emerging player in the progression of NAFLD. Nat. Rev. Gastroenterol. Hepatol..

[41] Horn P., Tacke F., Kloc M., Kubiak J.Z., Halasa M. (2024). Liver Macrophage Diversity in Health and Disease. Monocytes and Macrophages in Development, Regeneration, and Disease. Results and Problems in Cell Differentiation.

[42] Daemen S., Gainullina A., Kalugotla G., He L., Chan M.M., Beals J.W., Liss K.H., Klein S., Feldstein A.E., Finck B.N. (2021). Dynamic shifts in the composition of resident and recruited macrophages influence tissue re-modeling in NASH. Cell Rep..

[43] Tarantino G., Citro V. (2026). Liver-spleen axis: Deciphering a crucial crosstalk in non-alcoholic fatty liver disease progression and therapeutic implications. World J. Gastroenterol..

[44] Lv Y., Lau W.Y., Li Y., Deng J., Han X., Gong X., Liu N., Wu H. (2016). Hypersplenism: History and current status. Exp. Ther. Med..

[45] Tsushima Y., Endo K. (2000). Spleen enlargement in patients with nonalcoholic fatty liver: Correlation between degree of fatty infiltration in liver and size of spleen. Dig. Dis. Sci..

[46] Keramida G., Dunford A., Kaya G., Anagnostopoulos C.D., Peters A.M. (2018). Hepato-splenic axis: Hepatic and splenic metabolic activities are linked. Am. J. Nucl. Med. Mol. Imaging.

[47] Tarantino G., Citro V., Balsano C. (2021). Liver-spleen axis in nonalcoholic fatty liver disease. Expert Rev. Gastroenterol. Hepatol..

[48] Savastano S., Di Somma C., Pizza G., De Rosa A., Nedi V., Rossi A., Orio F., Lombardi G., Colao A., Tarantino G. (2011). Liver- spleen axis, insulin-like growth factor-(IGF)-I axis and fat mass in overweight/obese females. J. Transl. Med..

[49] Barrea L., Di Somma C., Muscogiuri G., Tarantino G., Tenore G.C., Orio F., Colao A., Savastano S. (2018). Nutrition, inflammation and liver-spleen axis. Crit. Rev. Food Sci. Nutr..

[50] Brummer C., Singer K., Renner K., Brussa C., Hellerbrand C., Dorne C., Reichelt-Wurmf S., Gronwaldg W., Pukropa T., Herra W. (2025). The spleen-liver axis supports obesity-induced systemic and fatty liver inflammation via MDSC and NKT cell enrichment. Mol. Cell. Endocrinol..

[51] Zhang S., Wan D., Zhu M., Wang G., Zhang X., Huang N., Zhang J., Zhang C., Shang Q., Zhang C. (2023). CD11b + CD43 hi Ly6C lo splenocyte-derived macrophages exacerbate liver fibrosis via spleen-liver axis. Hepatology.

[52] Zhang L., Wang Y., Wei K., Nie W., Feng Y., Shi Z., Xiao H., Xie W., Lin Y., Zeng X. (2026). Metabolically and epigenetically reprogrammed splenic TRNP1hiCD8+ T cells exacerbate liver fibrosis. Nat. Genet..

[53] Lee Y.A., Wallace M.C., Friedman S.L. (2015). Pathobiology of liver fibrosis: A translational success story. Gut.

[54] Parola M., Pinzani M. (2019). Liver fibrosis: Pathophysiology, pathogenetic targets and clinical issues. Mol. Asp. Med..

[55] Schwabe R.F., Tacke F., Sugimoto A., Friedman S.L. (2025). Antifibrotic therapies for metabolic dysfunction-assoiated steatotic liver disease. JHEP Rep..

[56] Trivedi P., Wang S., Friedman S.L. (2021). The power of plasticity-metabolic regulation of hepatic stellate cells. Cell Metab..

[57] Krenkel O., Hundertmark J., Ritz T.P., Weiskirchen R., Tacke F. (2019). Single cell RNA sequencing identifies subsets of hepatic stellate cells and myofibroblasts in liver fibrosis. Cells.

[58] Xiong X., Kuang H., Ansari S., Liu T., Gong J., Wang S., Zhao X.Y., Ji Y., Li C., Guo L. (2019). Landscape of intercellular crosstalk in healthy and NASH liver revealed by single-cell secretome gene analysis. Mol. Cell.

[59] Tanaka M., Miyajima A. (2003). Oncostatin M, a multifunctional cytokine. Rev. Physiol. Biochem. Pharmacol..

[60] Zarling J.M., Shoyab M., Marquardt H., Hanson M.B., Lioubin M.N., Todaro G.J. (1986). Oncostatin M: A growth regulator produced by differentiated histiocytic lymphoma cells. Proc. Natl. Acad. Sci. USA.

[61] Stephens J.M., Elks C.M. (2017). Oncostatin M: Potential Implications for Malignancy and Metabolism. Curr. Pharm. Des..

[62] Masjedi A., Hajizadeh F., Beigi Dargani F., Beyzai B., Aksoun M., Hojjat-Farsangi M., Zekiy A., Jadidi-Niaragh F. (2021). Oncostatin M: A mysterious cytokine in cancers. Int. Immunopharmacol..

[63] Caligiuri A., Gitto S., Lori G., Marra F., Parola M., Cannito S., Gentilini A. (2022). Oncostatin M: From intracellular signaling to therapeutic targets in liver cancer. Cancers.

[64] Wolf C.L., Pruett C., Lighter D., Jorcyk C.L. (2023). The clinical relevance of OSM in inflammatory diseases: A comprehensive review. Front. Immunol..

[65] Rose T.M., Bruce A.G. (1991). Oncostatin M is a member of a cytokine family that includes leukemia-inhibitory factor, granulocyte colony-stimulating factor, and interleukin 6. Proc. Natl. Acad. Sci. USA.

[66] Hermanns H.M. (2015). Oncostatin M and interleukin-31: Cytokines, receptors, signal transduction and physiology. Cytokine Growth Factor Rev..

[67] Di Maira G., Foglia B., Napione L., Turato C., Maggiora M., Sutti S., Novo E., Alvaro M., Autelli R., Colombatto S. (2022). Oncostatin M is overexpressed in NASH-related hepatocellular carcinoma and promotes cancer cell invasiveness and angiogenesis. J. Pathol..

[68] Giovannini M., Djabali M., McElligott D., Selleri L., Evans G.A. (1993). Tandem linkage of genes coding for leukemia inhibitory factor (LIF) and oncostatin M (OSM) on human chromosome 22. Cytogenet. Cell Genet..

[69] Jeffery E., Price V., Gearing D.A. (1993). Close proximity of the genes for leukemia inhibitory factor and oncostatin M. Cytokine.

[70] Yoshimura A., Ichihara M., Kinjyo I., Moriyama M., Copeland N.G., Gilbert D.J., Jenkins N.A., Hara T., Miyajima A. (1996). Mouse oncostatin M: An immediate early gene induced by multiple cytokines through the JAK-STAT5 pathway. EMBO J..

[71] Ma Y., Streiff R.J., Liu J., Spence M.J., Vestal R.E. (1999). Cloning and characterization of the human oncostatin M promoter. Nucleic Acids Res..

[72] Repovic P., Benveniste E.N. (2002). Prostaglandin E2 is a novel inducer of Oncostatin M expression in macrophages and microglia. J. Neurosci..

[73] Henkel J., Gärtner D., Dorn C., Hellerbrand C., Schanze N., Elz S.R., Püschel G.P. (2011). Oncostatin M produced in Kupffer cells in response to PGE2: Possible contributor to hepatic insulin resistance and steatosis. Lab. Investig..

[74] Neininger A., Kontoyiannis D., Kotlyarov A., Winzen R., Eckert R., Volk H.-D., Holtmann H., Kollias G., Gaestel M. (2002). MK2 targets AU-rich elements and regulates niosynthesis of tumor necrosis factor and Interleukin-6 independently at different post-transcriptional Levels. J. Biol. Chem..

[75] Linsley P.S., Kallestad J., Ochs V., Neubauer M. (1990). Cleavage of a hydrophilic C-terminal domain increases growth-inhibitory activity of oncostatin M. Mol. Cell. Biol..

[76] Somasundaram R., Ruehl M., Schaefer B., Schmid M., Ackermann R., Riecken E.O., Zeitz M., Schuppan D. (2002). Interstitial Collagens I, III, and VI Sequester and Modulate the Multifunctional Cytokine Oncostatin M. J. Biol. Chem..

[77] Ryan R.E., Martin B., Mellor L., Jacob R.B., Tawara K., McDougal O.M., Oxford J.T., Jorcyk C.L. (2015). Oncostatin M binds to extracellular matrix in a bioactive conformation: Implications for inflammation and metastasis. Cytokine.

[78] Bamber B., Reife R.A., Haugen H., Clegg C. (1998). Oncostatin M stimulates excessive extracellular matrix accumulation in a transgenic mouse model of connective tissue disease. J. Mol. Med..

[79] Wong S., Botelho F.M., Rodrigues R.M., Richards C.D. (2014). Oncostatin M overexpression induces matrix deposition, STAT3 activation, and SMAD1 Dysregulation in lungs of fibrosis-resistant BALB/c mice. Lab. Investig..

[80] Heinrich P.C., Behrmann I., Müller-Newen G., Schaper F., Graeve L. (1998). Interleukin-6-type cytokine signalling through the gp130/Jak/STAT pathway. Biochem. J..

[81] Grant S.L., Begley C.G. (1999). The oncostatin M signalling pathway: Reversing the neoplastic phenotype?. Mol. Med. Today.

[82] Heinrich P.C., Behrmann I., Haan S., Hermanns H.M., Schaper F. (2003). Principles of interleukin (IL)-6-type cytokine signalling and its regulation. Biochem. J..

[83] Böing I., Stross C., Radtke S., Lippok B.E., Heinrich P.C., Hermanns H.M. (2006). Oncostatin M-induced activation of stress-activated MAP kinases depends on tyrosine 861 in the OSM receptor and requires Jak1 but not Src kinases. Cell. Signal..

[84] Wang Y., Robledo O., Kinzie E., Blanchard F., Richards C., Miyajima A., Baumann H. (2000). Receptor subunit-specific action of oncostatin M in hepatic cells and its modulation by leukemia inhibitory factor. J. Biol. Chem..

[85] Smyth D.C., Kerr C., Richards C.D. (2006). Oncostatin M-induced IL-6 expression in murine fibroblasts requires the activation of protein kinase C delta. J. Immunol..

[86] Nurcis J., Foglia B., Rosso C., Provera A., Vecchio C., Maggiora M., Gambella A., Chianese U., Bocca C., Caviglia G.P. (2026). The role of OSM/OSMRβ axis in shaping the tumor microenvironment favoring MASLD-related HCC immune evasion. Hepatology.

[87] Wallace P.M., MacMaster J.F., Rouleau K.A., Brown T.J., Loy J.K., Donaldson K.L., Wahl A.F. (1999). Regulation of inflammatory responses by oncostatin M. J. Immunol..

[88] Wahl A.F., Wallace P.M. (2001). Oncostatin M in the anti-inflammatory response. Ann. Rheum. Dis..

[89] Mozaffarian A., Brewer A.W., Trueblood E.S., Luzina I.G., Todd N.W., Atamas S.P., Arnett H.A. (2008). Mechanisms of oncostatin M-induced pulmonary inflammation and fibrosis. J. Immunol..

[90] Ayaub E.A., Dubey A., Imani J., Botelho F., Kolb M.R.J., Richards C.D., Ask K. (2017). Overexpression of OSM and IL-6 impacts the polarization of pro-fibrotic macrophages and the development of bleomycin-induced lung fibro sis. Sci. Rep..

[91] Simpson J.L., Baines K.J., Boyle M.J., Scott R.J., Gibson P.G. (2009). Oncostatin m (osm) is increased in asthma with incompletely reversible airflow obstruction. Exp. Lung. Res..

[92] West N.R., Hegazy A.N., Owens B.M.J., Bullers S.J., Linggi B., Buonocore S., Coccia M., Görtz D., This S., Stockenhuber K. (2017). Oncostatin M drives intestinal inflammation and predicts response to tumor necrosis factor–neutralizing the rapy in patients with inflammatory bowel disease. Nat. Med..

[93] Guo A., Ross C., Chande N., Gregor J., Ponich T., Khanna R., Sey M., Beaton M., Yan B., Kim R.B. (2022). High oncostatin M predicts lack of clinical remission for patients with inflammatory bowel disease on tumor necrosis factor α antagonists. Sci. Rep..

[94] Hanlon M.M., Rakovich T., Cunningham C.C., Ansboro S., Veale D.J., Fearon U., McGarry T. (2019). STAT3 mediates the differential effects of oncostatinMand TNFa on RA synovial fibroblast and endothelial cell function. Front. Immunol..

[95] McGarry T., Orr C., Wade S., Biniecka M., Wade S., Gallagher L., Low C., Veale D.J., Fearon U. (2018). JAK/STAT blockade alters synovial bioenergetics, mitochondrial function, and proinflammatory mediators in rheumatoid arthritis. Arthritis Rheumatol..

[96] Plater-Zyberk C., Buckton J., Thompson S., Spaull J., Zanders E., Papworth J., Life P.F. (2001). Amelioration of arthritis in two murine models using antibodies to oncostatin M. Arthritis Rheumatol..

[97] Zhang X., Li J., Qin J.J., Cheng W.L., Zhu X., Gong F.H., She Z., Huang Z., Xia H., Li H. (2017). Oncostatin M receptor β deficiency attenuates atherogenesis by inhibiting JAK2/STAT3 signaling in macrophages. J. Lipid Res..

[98] Albasanz-Puig A., Murray J., Preusch M., Coan D., Namekata M., Patel Y., Dong Z.M., Rosenfeld M.E., Wijelath E.S. (2011). Oncostatin M is expressed in atherosclerotic lesions: A role for Oncostatin M in the pathogenesis of atherosclerosis. Atherosclerosis.

[99] van Keulen D., Pouwer M.G., Emilsson V., Matic L.P., Pieterman E.J., Hedin U., Gudnason V., Jennings L.L., Holmstrøm K., Nielsen B.S. (2019). Oncostatin Mreduces atherosclerosis development in APOE3Leiden.CETP mice and is associated with increased survival probability in humans. PLoS ONE.

[100] Zhang X., Zhu D., Wei L., Zhao Z., Qi X., Li Z., Sun D. (2015). OSM enhances angiogenesis and improves cardiac function after myocardial infarction. BioMed Res. Int..

[101] Hu J., Zhang L., Zhao Z., Zhang M., Lin J., Wang J., Yu W., Man W., Li C., Zhang R. (2017). OSM mitigates postinfarction cardiac remodeling and dysfunction by up-regulating autophagy through Mst1 suppression. Biochim. Biophys. Acta Mol. Basis Dis..

[102] David E., Guihard P., Brounais B., Riet A., Charrier C., Battaglia S., Gouin F., Ponsolle S., Bot R.L., Richards C.D. (2011). Direct anti-cancer effect of oncostatin M on chondrosarcoma. Int. J. Cancer.

[103] Lázár-Molnár E., Hegyesi H., Tóth S., Falus A. (2000). Autocrine and paracrine regulation by cytokines and growth Factors in melanoma. Cytokine.

[104] Miyajima A., Kinoshita T., Tanaka M., Kamiya A., Mukouyama Y., Hara T. (2000). Role of oncostatin M in hematopoiesis and liver development. Cytokine Growth Factor Rev..

[105] Znoyko I., Sohara N., Spicer S.S., Trojanowska M., Reuben A. (2005). Expression of oncostatin M and its receptors in normal and cirrhotic human liver. J. Hepatol..

[106] Levy M.T., Trojanowska M., Reuben A. (2000). Oncostatin M: A cytokine upregulated in human cirrhosis, increases collagen production by human hepatic stellate cells. J. Hepatol..

[107] Nakamura K., Nonaka H., Saito H., Tanaka M., Miyajima A. (2004). Hepatocyte proliferation and tissue remodeling is impaired after liver injury in oncostatin M receptor knockout mice. Hepatology.

[108] Okaya A., Kitanaka J., Kitanaka N., Satake M., Kim Y., Terada K., Sugiyama T., Takemura M., Fujimoto J., Terada N. (2005). Oncostatin M inhibits proliferation of rat oval cells, OC15-5, inducing differentiation into hepatocytes. Am. J. Pathol..

[109] Hamada T., Sato A., Hirano T., Yamamoto T., Son G., Onodera M., Torii I., Nishigami T., Tanaka M., Miyajima A. (2007). Oncostatin M gene therapy attenuates liver damage induced by dime thylnitrosamine in rats. Am. J. Pathol..

[110] Vollmer S., Kappler V., Kaczor J., Flügel D., Rolvering C., Kato N., Kietzmann T., Behrmann I., Haan C. (2009). Hypoxia-inducible factor 1α is up-regulated by oncostatin M and participates in oncostatin M signaling. Hepatology.

[111] Liang H., Block T.M., Wang M., Nefsky B., Long R., Hafner J., Mehta A.S., Marreroc J., Gishd R., Norton P.A. (2012). Interleukin-6 and oncostatin M are elevated in liver disease in conjunction with candidate hepatocellular carcinoma biomarker GP73. Cancer Biomark..

[112] Iftikhar R., Kladney R.D., Havlioglu N., Schmitt-Gräff A., Gusmirovic I., Solomon H., Luxon B.A., Bacon B.R., Fimmel C.J. (2004). Disease- and cell-specific expression of GP73 in human liver disease. Am. J. Gastroenterol..

[113] Reiner M.O., Stenner F., Liewen H., Soll C., Breitenstein S., Pestalozzi B.C., Samaras P., Probst-Hensch N., Hellerbrand C., Müllhaupt B. (2009). Golgi phosphoprotein 2 (GOLPH2) expression in liver tumors and its value as a serum mar ker in hepatocellular carcinoma. Hepatology.

[114] Sohara N., Trojanowska M., Reuben A. (2002). Oncostatin M stimulates tissue inhibitor of metalloproteinase-1 via a MEK-sensitive mechanism in human myofibroblasts. J. Hepatol..

[115] Matsuda M., Tsurusaki S., Miyata N., Saijou E., Okochi H., Miyajima A., Tanaka M. (2017). Oncostatin M causes liver fibrosis by regulating cooperation between hepatic stellate cells and macrophages in mice. Hepatology.

[116] Foglia B., Sutti S., Pedicini D., Cannito S., Bocca C., Maggiora M., Bevacqua M.R., Rosso C., Bugianesi E., Albano E. (2019). Oncostatin M, A Profibrogenic Mediator Overexpressed in Non-Alcoholic Fatty Liver Disease, Stimulates Migration of Hepatic Myofibroblasts. Cells.

[117] Sung P.S., Kim C.M., Cha J.H., Park J.Y., Yu Y.S., Wang H.J., Kim J.K., Bae S.H. (2022). A Unique Immune-Related Gene Signature Represents Advanced Liver Fibrosis and Reveals Potential Therapeutic Targets. Biomedicines.

[118] Melis M., Marino R., Tian J., Johnson C., Sethi R., Oertel M., Fox I.J., Locker J. (2024). Mechanism and Effect of HNF4alpha Decrease in a Rat Model of Cirrhosis and Liver Failure. Cell. Mol. Gastroenterol. Hepatol..

[119] Zhang X., Yang X., Sun X., Qin Q., Hou Y., Jia M., Su X., Chen Y. (2025). Yes-associated protein in hepatocytes protects against early alcohol-associated liver disease. Liver Int..

[120] Russell J.O., Camargo F.D. (2022). Hippo Signalling in the Liver: Rolein Development, Regeneration and Disease. Nat. Rev. Gastroenterol. Hepatol..

[121] Chen D., Zhang H., Zhang X., Xia S., Qin Q., Hou Y., Jia M., Chen Y. (2022). Roles of Yes-associated protein and transcriptional coactivator with PDZ-binding motif in non-neoplastic liver liseases. Biomed. Pharmacother..

[122] Komori T., Tanaka M., Senba E., Miyajima A., Morikawa Y. (2014). Deficiency of oncostatin M receptor β (OSMRβ) exacerbates high-fat diet-induced obesity and related metabolic disorders in mice. J. Biol. Chem..

[123] Luo P., Wang P.-X., Li Z.-Z., Xiao-Jing Zhang X.-J., Jiang X., Gong J., Qin J.-J., Guo J., Zhu X., Yang S. (2016). Hepatic oncostatin M receptor β regulates obesity-induced steatosis and insulin resistance. Am. J. Pathol..

[124] Han H., Ge X., Komakula S.S.B., Desert R., Das S., Song Z., Chen W., Athavale D., Gaskell H., Lantvit D. (2023). Macrophage-derived Osteopontin (SPP1) protects from nonalcoholic steatohepatitis. Gastroenterology.

[125] Nunez-Garcia M., Gomez-Santos B., Buque X., Garcìa-Rodrigues J.L., Romero M.R., Marin J.J.G., Arteta B., Garcìa-Monzon C., Castaño L., Syn W. (2017). Osteopontin regulates the cross-talk between phosphatidylcholine and cholesterol metabolism in mouse liver. J. Lipid Res..

[126] Song Z., Chen W., Athavale D., Ge X., Desert R., Das S., Han H., Nieto N. (2021). Osteopontin takes center stage in chronic liver disease. Hepatology.

[127] Grove R.I., Mazzucco C.E., Radka S.F., Shoyab M., Kiener P.A. (1991). Oncostatin M up-regulates low density lipoprotein receptors in HepG2 cells by a novel mechanism. J. Biol. Chem..

[128] Liu J., Shoyab M., Grove R.I. (1993). Induction of Egr-1 by oncostatin M precedes up-regulation of low density lipoprotein receptors in HepG2 cells. Cell Growth Differ..

[129] Liu J., Grove R.I., Vestal R.E. (1994). Oncostatin M activates low density lipoprotein receptor gene transcription in sterol-repressed liver cells. Cell Growth Differ..

[130] Vasse M., Paysant J., Soria J., Collet J.P., Vannier J.P., Soria C. (1996). Regulation of fibrinogen biosynthesis by cytokines, consequences on the vascular risk. Haemostasis.

[131] Haselmann J., Goppelt-Struebe M. (1997). Glucocorticoids inhibit oncostatin M-induced phospholipase A2 gene expression in human hepatoma cells. Cytokine.

[132] Cichy J., Rose-John S., Potempa J., Pryjma J., Travis J. (1997). Oncostatin M stimulates the expression and release of the IL-6 receptor in human hepatoma HepG2 cells. J. Immunol..

[133] Blanchard F., Tracy E., Smith J., Chattopadhyay S., Wang Y., Held W.A., Baumann H. (2003). DNA methylation controls the responsiveness of hepatoma cells to leukemia inhibitory factor. Hepatology.

[134] Pardo-Saganta A., Latasa M.U., Castillo J., Alvarez-Asiain L., Perugorría M.J., Sarobe P., Rodriguez-Ortigosa C.M., Prieto J., Berasain C., Santamaría M. (2009). The epidermal growth factor receptor ligand amphiregulin is a negative regulator of hepatic acute-phase gene expression. J. Hepatol..

[135] Castillo J., Erroba E., Perugorría M.J., Santamaría M., Lee D.C., Prieto J., Avila M.A., Berasain C. (2006). Amphiregulin contributes to the transformed phenotype of human hepatocellular carcinoma cells. Cancer Res..

[136] Yamashita T., Honda M., Nio K., Nakamoto Y., Yamashita T., Takamura H., Tani T., Zen Y., Kaneko S. (2010). Oncostatin m renders epithelial cell adhesion molecule-positive liver cancer stem cells sensitive to 5-Fluorouracil by inducing hepatocytic differentiation. Cancer Res..

[137] Kamiya A., Kinoshita T., Ito Y., Matsui T., Morikawa Y., Senba E., Nakashima K., Taga T., Yoshida K., Kishimoto T. (1999). Fetal liver development requires a paracrine action of oncostatin M through the gp130 signal transducer. EMBO J..

[138] Rolvering C., Zimmer A.D., Kozar I., Hermanns H.M., Letellier E., Vallar L., Nazarov P.V., Nicot N., Ginolhac A., Haan S. (2017). Crosstalk between different family members: IL27 recapitulates IFNγ responses in HCC cells, but is inhibited by IL6-type cytokines. Biochim. Biophys. Acta Mol. Cell. Res..

[139] Zhu C., Zhang R., Liu L., Rasool S.T., Mu Y., Sun W., Hao Q., Liu F., Zhu Y., Wu J. (2009). Hepatitis B virus enhances interleukin-27 expression both in vivo and in vitro. Clin. Immunol..

[140] Kao J.T., Feng C.L., Yu C.J., Tsai S.M., Hsu P.N., Chen Y.L., Wu Y.Y. (2015). IL-6, through p-STAT3 rather than p-STAT1, activates hepatocarcinogenesis and affects survival of hepatocellular carcinoma patients: A cohort study. BMC Gastroenterol..

[141] Prieto J., Melero I., Sangro B. (2015). Immunological landscape and immunotherapy of hepatocellular carcinoma. Nat. Rev. Gastroenterol. Hepatol..

[142] Rolvering C., Zimmer A.D., Ginolhac A., Margue C., Kirchmeyer M., Servais F., Hermanns H.M., Hergovits S., Nazarov P.V., Nicot N. (2018). The PD-L1- and IL6-mediated dampening of the IL27/STAT1 anticancer responses are prevented by α-PD-L1 or α-IL6 antibodies. J. Leukoc. Biol..

[143] Yang X., Shao C., Duan L., Hou X., Huang Y., Gao L., Zong C., Liu W., Jiang J., Ye F. (2021). Oncostatin M promotes hepatic progenitor cell activation and hepatocarcinogenesis via macrophage-derived tumor necrosis factor-α. Cancer Lett..

[144] Peng Z.P., Jiang Z.Z., Guo H.F., Zhou M.M., Huang Y.F., Ning W.R., Huang J.H., Zheng L., Wu Y. (2020). Glycolytic activation of monocytes regulates the accumulation and function of neutrophils in human hepatocellular carcinoma. J. Hepatol..

[145] Kuang D.M., Zhao Q., Wu Y., Peng C., Wang J., Xu Z., Yin X.-Y., Zheng L. (2011). Peritumoral neutrophils link inflammatory response to disease progression by fostering angiogenesis in hepatocellular carcinoma. J. Hepatol..

[146] Coffelt S.B., Wellenstein M.D., de Visser K.E. (2016). Neutrophils in cancer: Neutral no more. Nat. Rev. Cancer.

[147] Shigematsu Y., Tanaka K., Amori G., Kanda H., Takahashi Y., Takazawa Y., Takeuchi K., Inamura K. (2024). Potential involvement of oncostatin M in the immunosuppressive tumor immune microenvironment in hepatocellular carcinoma with vessels encapsulating tumor clusters. Hepatol. Res..

[148] Shigematsu Y., Kanda H., Takahashi Y., Takeuchi K., Inamura K. (2025). Relationships between tumor CD147 expression, tumor-infiltrating lymphocytes, and oncostatin M in hepatocellular carcinoma. Virchows Arch..

[149] Peng F., Li H., You Q., Li H., Wu D., Jiang C., Deng G., Li Y., Li Y., Wu Y. (2017). *CD*147 as a novel prognostic biomarker for hepatocellular carcinoma: A meta-analysis. BioMed Res. Int..

[150] Zhang Q., Zhou J., Ku X.M., Chen X.G., Zhang L., Xu J., Chen G.S., Li Q., Qian F., Tian R. (2007). Expression of CD147 as a significantly unfavorable prognostic factor in hepatocellular carcinoma. Eur. J. Cancer Prev..

[151] Li Y., Wu J., Zhang P. (2016). CCL15/CCR1 axis is involved in hepatocellular carcinoma cells migration and invasion. Tumor Biol..

